# In vitro models of the crosstalk between multiple myeloma and stromal cells recapitulate the mild NF-κB activation observed in vivo

**DOI:** 10.1038/s41419-024-07038-1

**Published:** 2024-10-06

**Authors:** Federica Colombo, Virginia Guzzeloni, Cise Kizilirmak, Francesca Brambilla, Jose Manuel Garcia-Manteiga, Anna Sofia Tascini, Federica Moalli, Francesca Mercalli, Maurilio Ponzoni, Rosanna Mezzapelle, Marina Ferrarini, Elisabetta Ferrero, Roberta Visone, Marco Rasponi, Marco E. Bianchi, Samuel Zambrano, Alessandra Agresti

**Affiliations:** 1 Division of Genetics and Cell Biology, IRCCS Ospedale San Raffaele, Milan, Italy; 2 Department of Electronics, Information and Bioengineering, Politecnico di Milano, Milan, Italy; 3 Division of Experimental Oncology, IRCCS Ospedale San Raffaele, Milan, Italy; 4 Universita‘ Vita-Salute San Raffaele, Milan, Italy; 5 Center for Omics Sciences, IRCCS Ospedale San Raffaele, Milan, Italy; 6 Division of Immunology, Transplantation and Infectious Diseases, IRCCS Ospedale San Raffaele, Milan, Italy; 7 Hospital “Maggiore della Carità”, Unit of Pathology, Novara, Italy; 8 B-Cell Neoplasia Unit, Division of Experimental Oncology, IRCCS Ospedale San Raffaele, Milan, Italy

**Keywords:** Cancer microenvironment, Chronic inflammation, Experimental models of disease

## Abstract

Multiple myeloma (MM) is linked to chronic NF-κB activity in myeloma cells, but this activity is generally considered a cell-autonomous property of the cancer cells. The precise extent of NF-κB activation and the contributions of the physical microenvironment and of cell-to-cell communications remain largely unknown. By quantitative immunofluorescence, we found that NF-κB is mildly and heterogeneously activated in a fraction of MM cells in human BMs, while only a minority of MM cells shows a strong activation. To gain quantitative insights on NF-κB activation in living MM cells, we combined advanced live imaging of endogenous p65 Venus-knocked-in in MM.1S and HS-5 cell lines to model MM and mesenchymal stromal cells (MSCs), cell co-cultures, microfluidics and custom microbioreactors to mimic the 3D-interactions within the bone marrow (BM) microenvironment. We found that i) reciprocal MM-MSC paracrine crosstalk and cell-to-scaffold interactions shape the inflammatory response in the BM; ii) the pro-inflammatory cytokine IL-1β, abundant in MM patients’ plasma, activates MSCs, whose paracrine signals are responsible for strong NF-κB activation in a minority of MM cells; iii) IL-1β, but not TNF-α, activates NF-κB in vivo in BM-engrafted MM cells, while its receptor inhibitor Anakinra reduces the global NF-κB activation. We propose that NF-κB activation in the BM of MM patients is mild, restricted to a minority of cells and modulated by the interplay of restraining physical microenvironmental cues and activating IL-1β-dependent stroma-to-MM crosstalk.

## Introduction

Multiple myeloma (MM) is a haematological malignancy characterized by the accumulation of malignant plasma cells in the bone marrow (BM) microenvironment (ME). Inflammation within BM niches is now increasingly recognized as a facilitating condition for malignant haematopoietic cell survival, expansion, and disease progression [1–3]. Importantly, inflammation and high IL-1β blood levels [4, 5] in MM patients persist after chemotherapy, and drive cancer resistance and relapse [6]. NF-κB, the major regulator of inflammation, has been broadly described as hyperactive in myeloma cells where it critically contributes to survival, proliferation, and resistance to therapy [7, 8]. NF-κB activation in MM cells has been inferred from transcriptomic data [9], immunohistochemistry (IHC) imaging on BM samples [10, 11] or in vitro by biochemical approaches on isolated cell populations, in experimental timeframes of days [11, 12].

Mesenchymal stromal cells (MSCs) are instrumental for MM progression and chemoresistance, and NF-κB is reported to be activated in vitro by feed-forward signals from both myeloma and other BM-dwelling cell types [12, 13]. A recent paper described a large subpopulation of inflammatory MSC (iMSCs) in the BM of myeloma patients, corresponding to 35–40% of the total stromal population and virtually absent in healthy subjects [14]. The transcriptional landscape of iMSCs predicts NF-κB activation likely due to cytokines and chemokines (CKs), such as IL-1β and TNFα, secreted by macrophages and T lymphocytes. The authors proposed that the paracrine iMSC-MM crosstalk would shape the overall activity of ME components. However, a thorough characterization of the activation dynamics and NF-κB regulation in living MM cells and MSCs in the BM microenvironment is missing. A deeper understanding of these mechanisms would be instrumental to target NF-κB to reduce overall tumor-promoting BM inflammation and increase chemotherapy efficacy [15].

Here, we found that most MM cells dwelling in the BM of both mice and humans show a mild NF-κB activation, whereas only in few cells NF-κB is highly activated. These findings contrast with the prevailing view in the field, but still indicate that low-level NF-κB activation might be shaped by the BM microenvironment. We thus aimed at understanding the origin of NF-κB activation in this context.

We [16, 17] and others [18, 19] recently showed that NF-κB activity in living cells consists of cycles of nuclear-to-cytoplasm localizations (oscillations) that are highly heterogeneous and asynchronous at cell population level. Being a transcription factor, NF-κB drives transcription only when localized in the nucleus. It follows that NF-κB driven transcription rises and falls over time. Using simple system perturbations, we obtained dynamical patterns of NF-κB activity that could be connected to specific NF-κB driven transcriptional outputs [16]; this cannot be obtained in a static experimental setup [20, 21]. Hence, we decided to use such a dynamic approach to shed light on the mechanisms leading to NF-κB activation within cancer tissues and on cells in response to complex inflammatory signals in the ME.

To provide a reliable characterization of NF-κB activity in myeloma cells at single-cell level, we exploited an integrated approach comprising MSCs and MM cells that express fluorescently tagged endogenous p65/NF-κB, high throughput live imaging, microfluidic devices, mathematical modelling, and bioinformatics.

In vitro experiments showed that MSCs and MM cells are sensitive to reciprocal crosstalk, which establishes a basal activity of NF-κB in MM cells in crowded environments. IL-1β, an inflammatory cytokine detected at high levels in the patient’s blood [4, 5], powerfully triggers NF-κB in MSCs, but not in MM cells; however, the paracrine secretion it induces in MSC cells strongly activates a fraction of the MM cell population.

At transcriptional level, a short-term paracrine crosstalk is sufficient to trigger the reprogramming of a small fraction of MM cells and MSCs toward a more malignant or inflammatory phenotype, respectively. In a mouse model, a short 3 h treatment with IL-1β, but not with TNF-α, activates NF-κB in a fraction of BM MM cells, while the IL-1β inhibitor Anakinra reduces their level of NF-κB activation.

Overall, our in vitro and in vivo models suggest that the prognostic NF-κB inflammatory signature in MM described in previous studies [9] is triggered by IL-1β on MSCs, which induce NF-κB activation in MM cells.

## Results

### NF-κB activation in BM-dwelling MM cells is mild and heterogeneous

*W*e first evaluated the abundance of p65/NF-κB protein in the broad and varied collection of IHC images of human tumours at different stage of progression that are stored in the Protein Atlas (https://www.proteinatlas.org). Image browsing at tissue level highlighted p65 overexpression in tumor tissues as compared to healthy and tumor-peripheral tissues. Historically, high NF-κB expression has been frequently interpreted as overactivation, but NF-κB is transcriptionally active only when present into the nucleus. Indeed, in most of the Protein Atlas images [22], p65 staining is very low in the nuclei and mainly localises in the cytoplasm, suggesting a moderate NF-κB activity.

**Fig. 1:**
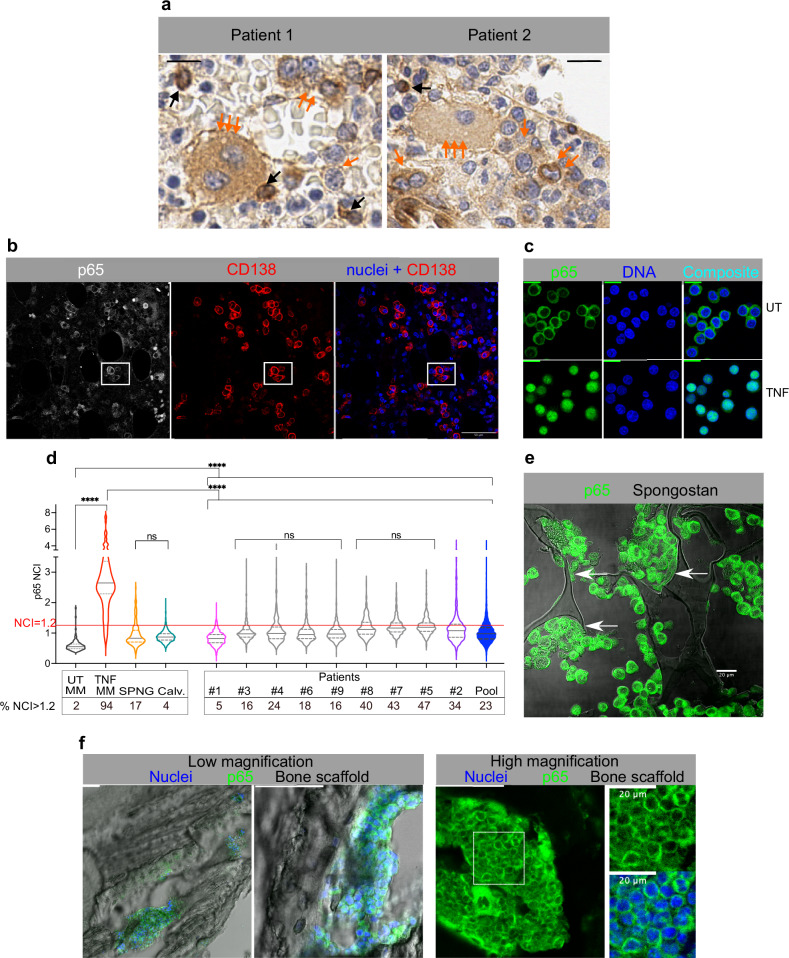
NF-κB activation in BM-dwelling MM cells is mild and heterogeneous. **a** NF-κB localizes in the cytoplasm of MM cells in patients’ BMs. Formalin Fixed Paraffin Embedded (FFPE) BM sections (Patient 1 and Patient 2) stained for p65 (DAB, brown signal) and counterstained with Haematoxylin (Blue). Orange arrows: a “set of three arrows” points to very large polynucleated cells. A “set of two” points to MM cells with dark brown cytoplasmic staining. Single arrows point to medium size MM cells with cytoplasmic staining and empty nuclei. Black arrows point to the few cells with heavy nuclear staining. Scale bar: 40 µm. **b** NF-κB localizes in the cytoplasm of CD138^+^ MM cells in myeloma patients’ BMs by IF. Confocal images of FFPE BM sections from myeloma patients stained for p65 (grey) and CD138 (red). Nuclei are counterstained with Hoechst 33342 (blue). The last panel is the composite image obtained by merging CD138 and nuclear fluorescent signals. Scale bar: 100 µm. Enlargements of the white square area in Supplementary Fig. 1d. **c** p65 localization by IF in human MM.1S cells. Cells untreated (UT) or treated with TNF-α for 60 min are stained for p65 (green) and nuclear DNA (blue). The last panel is the composite image. Scale bar: 20 µm. **d** Human MM cells in BMs show low levels of nuclear p65. Violin plots compare p65 NCI value distributions in CD138^+^ MM cells in the BM of nine MM patients (Pt.1-9 plus the pool of all the patients, full blue) with those from MM.1S cells either in static cultures (UT: unstimulated, TNF-α treated for 60’, pool of three experiments), or grown in coculture with HS-5 on Spongostan (SPNG, pool of two experiments), or engrafted in mouse calvaria (calvaria, pool of four mice). Median value: solid line, 25 and 75%: dashed lines. Numbers reported below the x-axis show the fraction (%) of cells displaying a p65 NCI > 1.2. Statistical analyses by ANOVA with Bonferroni multiple comparison test: significance as indicated in the plot: ****p < 0.0001, ns: not significant. **e** NF-κB is mainly cytoplasmic in MM.1S cells grown on SPNG scaffold in a RCCS Bioreactor. Composite confocal images for p65 (green) and bright field image (grey) on FFPE SPNG sections. Arrows point to stromal HS-5 cells (scale bar: 20 µm). Images are from one representative experiment out of three performed. **f** NF-κB is mainly cytoplasmic in MM.1S cells engrafting the mouse calvaria bones. Confocal images of MM.1S cells engrafted in mouse calvaria bones and IF stained for p65. Representative staining from 2 calvaria out of 7 analysed. Nuclei in blue, p65 in green, bone structures in grey, shown as composite images. Left: Large- and medium scale images of MM.1S cell clumps adhering to surface in calvaria cavities, scale bar 100 µm. Right: p65 staining image from a different calvaria section. The region identified by the white square is enlarged on the right and green and green plus blue images shown; scale bar: 100 µm, or 20 µm as indicated. Sample numerosity, median values and samples size above the NCI threshold are reported in Supplementary Table 3.

However, the Protein Atlas collection lacks images of myeloma BM samples stained for p65. We then verified the p65 localization in MM cells in the BM of two treatment-naïve MM patients (Pt.1 and Pt.2). Sections were stained for p65 with two different antibodies by both IHC and IF (Fig. 1a, and Fig. 1b, Supplementary Fig. 1a-f). As suggested by the pattern of p65 expression in other tumours present in the Protein Atlas, and unlike normal skin tissue (Supplementary Fig. 1a), both IHC and IF staining revealed a strong p65 staining in MM cells, with a prevalent cytoplasmic localization, sometimes forming an intense ring at the boundary between the nucleus and the thin cytoplasm **(**Fig. 1a, b, and Supplementary Fig. 1b–d). This finding suggests that p65/NF-κB is highly expressed in MM cells as compared to the surrounding BM cells. Approximately 5% of MM cells showed nuclear NF-κB staining. These data indicate heterogeneous NF-κB activation in MM cells (Fig. 1a, black arrows).

We then reinforced these preliminary findings by staining p65 in BM biopsies from seven more treatment-naïve myeloma patients, with different age, degree of plasma cell infiltration and disease stage (Supplementary Fig. 1 part 2 and Supplementary Information file). As in the first two samples, the additional seven samples showed an overall high p65 staining in the cytoplasm of cells positive for CD138, a specific membrane marker for MM cells, and a low but detectable staining in the nucleus.

To perform unbiased comparisons, we rigorously quantified nuclear p65 levels at single cell level by calculating the p65 “Nuclear to Cytoplasmic Intensity” ratio (NCI) [16, 23]. This is an internally normalized descriptor of NF-κB distribution which indicates the degree of NF-κB/p65 nuclear localization [16, 23] and is not affected by the overall p65 amount. Therefore, it allows direct comparisons of p65 relative nuclear localization in different cell types and in different experiments or treatments.

We then compared the NF-κB activation in patients’ MM cells with that of a panel of MM cell lines, including MM.1S, U266, OPM2 and RPMI8226, primary B cells and the reference JY B cell line. Nuclear NF-κB levels in untreated (UT) cells were similar in all these cell lines, while responses to TNF-α were heterogeneous; some cell lines died after 1-2 h of stimulation (Supplementary Fig. 4 panel b). Such responses might be ascribed to the combinatorial effect of mutations and Copy Number Variations for NF-κB regulatory genes (Supplementary Fig. 2a) in the various MM cell lines. Indeed, our mathematical model [24] does predict NF-κB atypical dynamic responses as a consequence of different genetic alterations, like the monoallelic deletion of the *NFKBIA* gene borne by MM.1S cells, Gain-of-Function mutations in IKK kinases or Loss-of-Function mutations in NF-κB repressors (Supplementary Fig. 2b, c).

Considering the variable NF-κB response and sensitivity to TNF-α, we decided to focus on MM.1S cells, which have a minimal mutational burden (Supplementary Table 1), do not die upon stimulation, and show a robust NF-κB activation upon inflammatory stimuli in vitro. The MM.1S cell line is a well-established, reliable, and representative human myeloma model [25] that is widely used to study inflammatory cytokines [26], interactions with stromal cells [26] and apoptosis [12], and can engraft the BM of NSG mice within a few days [27]. Further data supporting our choice are reported in the Supplementary Information file related to Fig. 1 and Supplementary Figs. 2, 3, 4).

MM.1S cells showed a low but non-zero p65 signal in the nucleus when untreated, while TNF-α induced a strong p65/NF-κB nuclear re-localization (Fig. 1c). The p65 NCI was then compared between MM.1S cells and patients’ CD138 + MM cells in BM sections (Fig. 1b and Supplementary Fig. 1, part 2**)**. MM.1S cells had a median NCI value of 0.5 when untreated and of 2.6 during peak activation with 10 ng/ml TNF-α. Using a p65 NCI of 1.2 as a threshold for clear NF-κB activation, 2% of untreated cells and 94% of treated cells were activated. CD138^+^ MM cells from the nine patients showed p65 NCI values higher than those in untreated MM.1S cells, indicative of a mild activation. Activation in all patients was significantly lower than the activation induced by TNF-α in MM.1S cells, with median NCI values close to 1 and a fraction of activated cells ranging from 5 to 47% (NCI > 1.2). The violin plot in Fig. 1d summarises the p65 distributions of the samples analysed.

Overall, p65/NF-κB in MM cells in patients’ BMs is activated mildly and only in a fraction of cells, in partial contrast to the predominant description in the literature.

### In vitro and in vivo 3D MM cell cultures recapitulate the mild p65 activation in human BMs

To experimentally reproduce in vitro the peculiar NF-κB NCI distribution found in patients’ MM cells, we exploited a controllable Rotary Cell Culture System (RCCS) Bioreactor, where cells were grown for 24 h on 3D gelatin scaffolds that mimic the BM matrix [28]. In this simplistic but reproducible model of the BM, MM cells are modelled by the MM.1S cell line while the stromal component is represented by HS-5 cells, which faithfully recapitulate the transcription profiles, tumour biology and immune responses of primary MSCs [29]. Patients’ mesenchymal cells were excluded because of the imprint they might have received in the BM. MSCs from healthy donors’ BM were excluded because isolation procedures led to NF-κB activation.

Scaffolds were sectioned and IF stained for p65 (Fig. 1e). p65 NCI distribution was similar to those in patients, suggesting that 3D gelatin scaffolds could be a simple but reliable model to study the interactions between MM cells and their ME (Fig. 1d).

We also used an animal model to explore if it recapitulated the heterogeneous and mild NF-κB activation found in human BMs. MM.1S cells were intravenously (i.v.) injected in immunocompromised NSG mice (which have no B and T cells, and low macrophage and neutrophil counts). After 20 days, calvaria and femurs were analysed by immune staining and high-resolution confocal imaging (Fig. 1f). MM.1S cells filled or lined the BM niches as previously described by Intravital Microscopy [30]. On visual inspection, MM.1S nuclei appeared devoid of p65, which mainly localised in the cytoplasm (Fig. 1f, right). NCI quantifications (Fig. 1d) showed that NF-κB activation in MM.1S cells in the mouse model was significantly higher than in untreated MM.1S cells in vitro, with a fraction of activated cells (NCI > 1.2, Supplementary Fig. 1e).

Overall, our bioreactor and mouse MM models reproduced the heterogenous and mild NF-κB activation in MM patients. This indicates that NF-κB activation in the BM is mediated at least in part by cell-to-cell interactions, since the same cells in vitro are much less activated. Hence, we decided to investigate in depth the origin of this mild and heterogenous activation using MM.1S and HS-5 cells as models of MM and MSC cells, respectively.

### NF-κB dynamics in living MM cells are heterogeneous and remain active for hours after stimulation

Since our previous work highlighted how NF-κB dynamic behaviours can produce specific transcription profiles, we used real-time single-cell live imaging as the starting point to understand the pattern of NF-κB activation in the patients’ BMs.

**Fig. 2:**
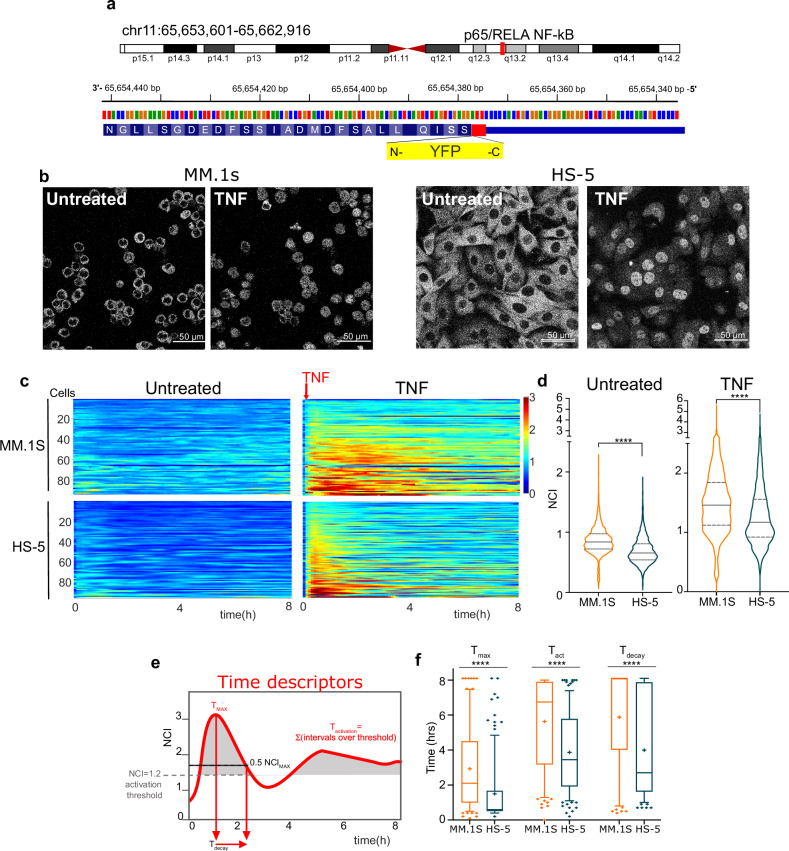
Heterogenous NF-κB dynamics in living MM cells are suggestive of autocrine/paracrine NF-kB modulation. **a** YFP knock-in cloning strategy. The Open Reading Frame of the Venus protein (YFP, yellow box) has been inserted upstream of the stop codon 552 (red) in exon 11. Genomic coordinates in the Methods section. **b** Live imaging of p65-YFP MM1.S cells and stromal HS-5. Confocal live cell images of unstimulated and 60’-TNF-α treated MM.1S (left) and HS-5 (right) cells after sorting. Scale bar: 50 µm. **c** Heterogenous NF-κB dynamics in living MM.1S cells and HS-5. Colorplots show NF-κB dynamics represented as NCI calculated every 6 min for 8 h in both untreated and TNF-α treated MM.1S cells and HS-5 cells, as indicated. Each line represents NCI values in a single cell as a function of time. The red arrow indicates the timepoint for TNF-α addition. y-axis: number of cells; x-axis: time in hours. Colorbar: NCI values from 0 (blue, cytoplasmic p65) to 3 (red, mainly nuclear p65). **d** Quantification of NF-κB dynamics in living MM.1S and HS-5 cells. Violin plots of NCI value distributions from the experiment in (**c**), representative of three performed. Median value: solid line, 25 and 75%: dashed lines. Significance between NCI distributions assessed by unpaired, two-tailed non-parametric *t*-test (Mann-Whitney*,* ****P < 0.0001,. N° of cells and Median values are reported in Supplementary Table 3. **e** Time parameters are needed to describe complex time-dependent dynamics. Time parameters are reported on an NCI plot from a single representative MM cell (red curve) stimulated for 8 h with TNF-α. T_max_ and T_act_ are indicated by red arrows. The dotted black line indicates 0.5-fold NCI_max_ to calculate T_dec_. **f** Time responses of p65 in MM cells are prolonged as compared in HS-5. Boxplots for T_max_, T_act_ and T_dec_ values upon TNF-α stimulation, reported for the experiment shown in Fig. 1c. Median value as a solid line, the “+” indicates the mean value. Statistical analyses: ANOVA+ Bonferroni test.

We engineered MM.1S and HS-5 cells with CRISPR/Cas9 to fuse the yellow Venus Fluorescent Protein coding region [31, 32] in frame with the endogenous p65 gene (hereafter p65-YFP, Fig. 2a and Supplementary Fig. 6a). Thanks to the endogenous promoter, this configuration allows the expression of physiological levels of active YFP-tagged p65 [17]. To our knowledge, this is the first example of stable genetic knock-in in MM cells, that per se are very resistant to experimental genetic manipulations. We also tried to engineer the MM cell lines shown in Supplementary Fig. 4, with no success; all modified cells died. In contrast, we successfully engineered several other non-MM cell types to express p65-YFP (Supplementary Fig. 5), indicating that myeloma cells are intrinsically sensitive to cytoplasmic DNA and/or lentiviral infection.

After flow cytometry sorting and expansion in culture, engineered MM.1S and HS-5 cells showed rather homogeneous p65-YFP intensity at basal level (Supplementary Fig. 6a, right). To quantify both basal dynamics and the responses of endogenous p65 to physiologic inflammatory stimuli [33], p65-YFP MM.1S and p65-YFP HS-5 cells were exposed or not to 10 ng/ml TNF-α in static cultures and imaged by time-lapse confocal microscopy for 8–12 h (Fig. 2b) in a controlled environment. Nuclear-to-cytoplasm dynamics of p65-YFP/NF-κB (NF-κB dynamics, in short) were recorded in hundreds of cells by calculating NCI every 6 minutes [16] for 6 to 8 h (Supplementary Fig. 6b, c). Experimental time windows in the hour range are commensurate with the duration of TNF-α driven classical NF-κB activation and exclude any possible superimposition of uncontrollable secondary and tertiary effects of disparate origins, either intrinsic or extrinsic, like non-canonical NF-κB activities, which arise upon stimulations of 16–24 h [34, 35]. Furthermore, NF-κB dynamics observed after TNF-α stimulation by immunofluorescence are comparable in wildtype and engineered cells (Supplementary Fig. 6d and Supplementary Information file).

Collective NF-κB dynamics in untreated MM.1S and HS-5 cells from a representative experiment are visualised by colorplots (Fig. 2c) where each line reports NCI values in a single cell along time according to a colour scale (blue for low NCI, and red for high NCI). Each experiment was replicated at least three times and the experimental reproducibility is reported in Supplementary Fig. 7a. Even at first glance, colorplots for unstimulated MM.1S cells showed detectable non-zero p65 activation levels (yellow tinges) that fluctuate over the 8 h acquisition (Fig. 2c, left), suggestive of a leaky negative feedback loop and/or auto/paracrine NF-κB activation. These dynamics are in accordance with the “inflammatory signature” described in MM primary cells and cell lines [9]. Basal p65 NCI in HS-5 cells is lower (blue prevalence) (Fig. 2c). TNF-α treatment in MM.1S cells induces a slow but strong, heterogeneous, and asynchronous NF-κB activation dynamics (Fig. 2c, right). NCI values stay high for many hours or even do not return to basal levels, in accordance with biochemical data and mathematical modelling in the literature [36] and our own (see Supplementary Fig. 2d). Conversely, a large fraction of HS-5 cells responds rather strongly and sharply to TNF-α with patterns of oscillatory dynamics, as shown for mouse embryonic fibroblasts [16, 17] and observed for several other cell lines (Supplementary Fig. 5).

As suggested by the colorplots, the time dimension strongly impacts on the global NF-κB activation; thus, we also calculated the time-integrated p65 activity (AUC, area under the curve, Supplementary Fig. 6f), which mirrors NCI distributions (Fig. 2d).

To compare the dynamics in different cells and conditions, we used various temporal descriptors (Fig. 2e). The time to the maximum (T_max_) suggests that NF-κB activation in response to TNF-α is more than two-fold faster in stromal than in MM.1S cells. In addition, in stromal cells the signal decay (T_dec_) is shorter and total activation time (T_act_) of NF-κB is less than 6 h. In contrast, NF-κB responses in myeloma cells stay above the activation threshold for a longer time with almost no decay after 8 h (Fig. 2f). Unsupervised k-means clustering of single cell tracks identify three subpopulations with different activation and decay kinetics. Of note, approximately 20% of myeloma cells responds more strongly than the average (Supplementary Fig. 7b–d).

Taken together, our analyses reveal that NF-κB dynamics in myeloma cells is different compared to both HS-5 and other previously reported cell types [18, 37, 38] The high basal levels in MM.1S cells are suggestive of autocrine/paracrine NF-κB modulation [9] at population level [39], possibly determined by a minority of highly activated cells that might signal to the rest of the population (Supplementary Fig. 7). The presence of autocrine/paracrine secretion makes it difficult to untangle the cell-autonomous from the cell-extrinsic contributions to NF-κB responses in myeloma cells. We thus reasoned that the removal of secreted molecules would allow to disentangle cell-autonomous from environment-driven NF-κB responses.

#### Microfluidic cultures highlight autocrine/paracrine modulation of NF-κB dynamics

**Fig. 3:**
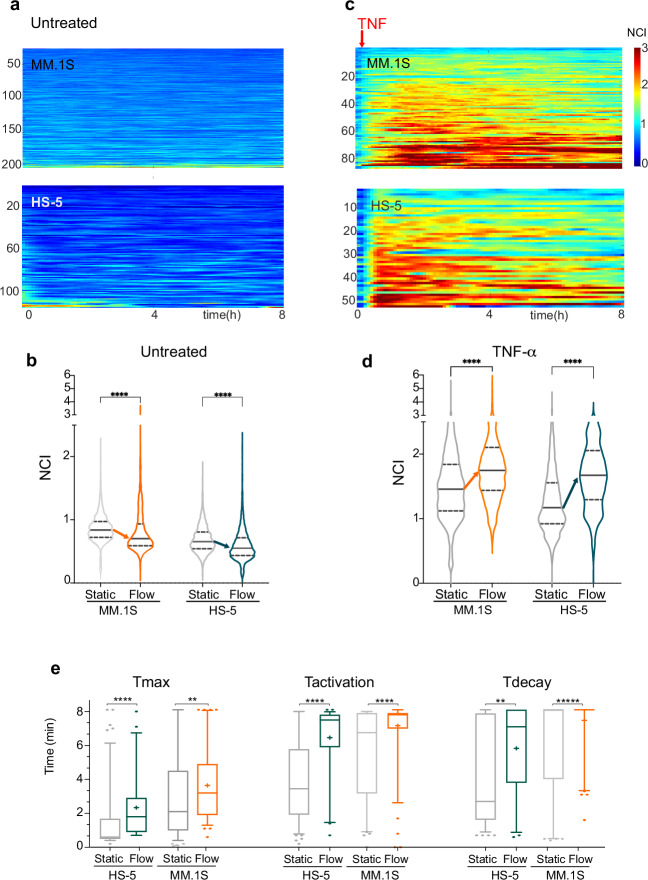
Autocrine/paracrine signalling modulates NF-κB dynamics in both HS-5 stromal and myeloma MM.1S cells. **a** Culture medium flow decreases NF-κB basal activity in both MM.1S cells and HS-5. Colorplots show basal NF-κB dynamics (NCI) for untreated myeloma cells and stromal cells for 8 h as indicated. Plots from a single experiment are representative of the four performed. y-axis: number of cells; x-axis: time in hours. NCI values colorbar from 0 (blue, cytoplasmic) to 3 (red, nuclear). **b** Quantification of basal NF-κB dynamics in cells exposed to medium flow. Violin plots show NCI values distributions in untreated MM.1S and HS-5 cells in static (grey) versus flow culture conditions (in colour) for 8 h in the experiment shown in “a”. Arrows indicate the decrease of the median value. Median value: solid line, 25 and 75%: dashed lines. Significance between NCI distributions was assessed by unpaired, two tailed non-parametric *t*-test (Mann-Whitney, *P* < 0.001, *** *P* < 0.0001, ****). N° of cells and Median values are reported in Supplementary Table 3. **c** Medium flow amplifies NF-κB responses to TNF-α in both MM.1S and HS-5 cells. Colorplots show NF-κB dynamics (NCI) for 8 h TNF-α treated MM.1S and HS-5 cells. y-axis: number of cells; x-axis: time in hours. Representative experiment of the four experiments performed. NCI values colorbar from 0 (blue, cytoplasmic) to 3 (red, nuclear). The red arrow indicates the timepoint for TNF-α addition. **d** Quantification of NF-κB dynamics in cells exposed to medium flow delivering TNF-α. Violin plots show NCI values distributions for TNF-α treated MM.1S and HS-5 cells in static (grey) versus flow culture conditions (colour) for 8 hours for the experiment represented in **c**. Arrows indicate the increase in median values. Median value: solid line, 25 and 75%: dashed lines. Significance between NCI distributions was assessed by unpaired, two tailed non-parametric t-test (Mann-Whitney, *P* < 0.001: ***, *P* < 0.0001: ****). N° of cells and Median values are reported in Supplementary Table 3. **e** The increase of T_act_ and T_dec_ defines the overall NF-κB activation increase upon TNF-α flow. Boxplots compare distributions for time descriptors in NF-κB dynamics in static and flow cultures for untreated or TNF-α treated cells as indicated. Median value drawn as a line, “+” indicates the mean value. Significance for T distribution differences was assessed by unpaired, two tailed non-parametric t-test (Mann-Whitney, *P* < 0.0001, **** *P* < 0.001, ***).

To dissect the contribution of autocrine-paracrine signalling in NF-κB dynamics for our models of MM and MSCs, we exploited a highly controllable microfluidic device in which a constant and gentle medium flow continuously removes secreted molecules [16] from the cell cultures (Supplementary Fig. 8a–c). Upon medium flow, the non-zero basal NF-κB activation state in MM.1S cells is attenuated as compared to static cultures (Fig. 3a, b). Rather unexpectedly, the same happened to HS-5 cells upon flow (Fig. 3a, b), suggesting that autocrine/paracrine signalling in both cell types impacts basal NF-κB activation. Instead, the degree of confluency appeared uninfluential on NF-κB dynamics (Supplementary Fig. 9).

On the other hand, flow-delivered TNF-α induced an almost synchronous p65-YFP nuclear translocation and a stronger p65 activation compared to static conditions in both MM.1S cells and stromal cells (Fig. 3c, d). In both cell types, p65 stayed active for longer periods (T_act_) with longer decay times (T_dec_) (Fig. 3e). Similarly to what described for static cultures, unsupervised k-means clustering of single cell tracks upon TNF-α identified a small MM.1S subpopulation (approx. 10%) that responds much more strongly to TNF-α than the average population (Supplementary Fig. 10).

Hence, our flow cultures allowed us to describe the NF-κB behaviour in the two living cell types both at basal conditions and in response to TNF-α. Results show that cell-autonomous and autocrine/paracrine activation co-exist and interact, leading HS-5 and MM.1S cells to respond differently in static environments as opposed to environments with high exchange rates. These results prompted us to first validate the presence of autocrine/paracrine signalling in static UT and TNF-α treated cells and then to analyse a possible MM-MSC crosstalk.

#### A molecular crosstalk between MSCs and MM cells tunes NF-κB responses

**Fig. 4:**
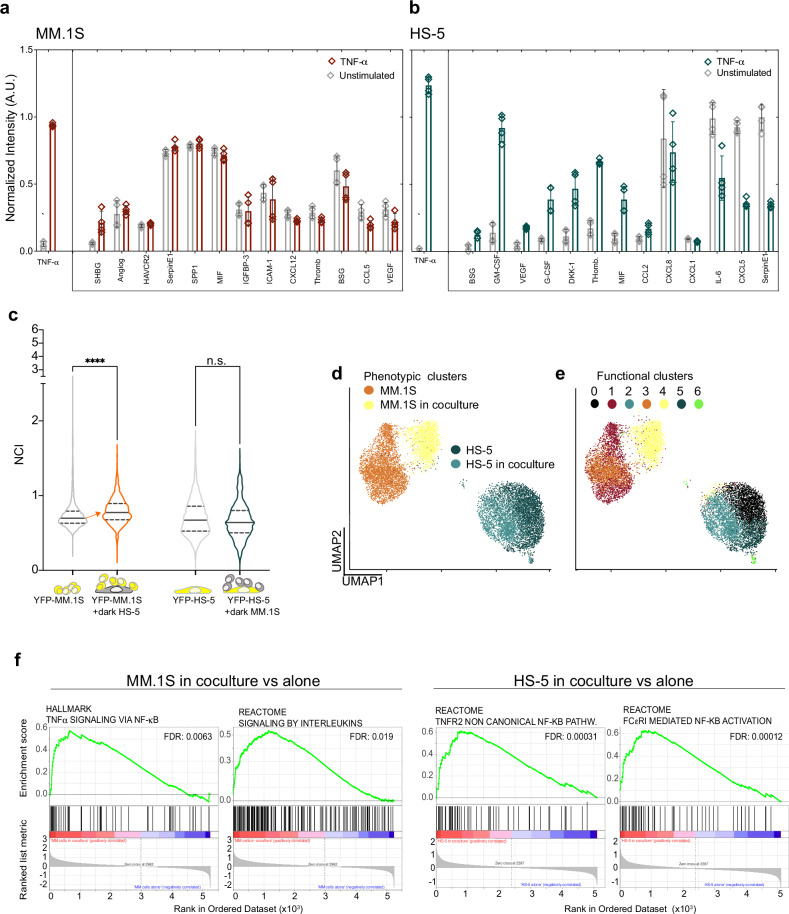
Cell-to-cell crosstalk is due to released chemokines and cytokines (CKs). **a** TNF-α driven secretory profiles in MM.1S cells. Histogram represents CK quantification in supernatants from untreated (grey bars) or TNF-α stimulated MM.1S cells (dark red) for two hours averaged from two independent experiments with technical duplicates. Error bars: SD. The quantification of the total TNF-α before and after TNF-α stimulation is plotted in a separated box on the left. y-axis: normalised fluorescence intensity in Arbitrary Units (AU). x-axis: detected CKs. **b** TNF-α driven secretory profiles in HS-5 cells. Histogram represents CK quantification in supernatants from untreated (grey bars) or TNF-α stimulated HS-5 cells (dark green) for two hours averaged from two independent experiments with technical duplicates. The quantification of the total TNF-α before and after TNF-α stimulation is plotted in a separated box on the left. The same experiment treating HS-5 with IL-1β is shown in Supplementary Fig. 11e. y-axis: normalised fluorescence intensity in AU. x-axis: detected CKs. Error bars: SD. **c** Paracrine signalling activates NF-κB dynamics only in MM.1S cells upon cocultures with HS-5 cells. Left: violin plots compare NF-κB NCI distributions in p65-YFP MM.1S cells alone (grey) or in coculture with wildtype HS-5 (red). Right: NF-κB NCI distributions in p65-YFP HS-5 alone (grey) or cocultured with wildtype MM.1S cells (green). The schematic on the bottom summarises the cultures: yellow cells express p65-YFP and the grey ones (dark) are wildtype. The orange arrow indicates the median NCI increase. y-axis: NCI. This experiment is representative of two performed. Significance between NCI distributions were assessed by ANOVA/Kruskal-Wallis test with Dunn’s correction for multiple comparisons (*P* < 0.0001 and ns). N° of cells and Median values are reported in Supplementary Table 3. **d** The transcription profile of MM.1S cells changes upon coculture with HS-5 cells. Single cell RNA-seq was performed on MM.1S and HS-5 cells, grown in monoculture or in coculture for 2 h. Dot-plot: UMAP clustering on cell type IDs identifies four populations represented as four coloured clusters (each dot is a single cell). MM.1S in monoculture, orange, in coculture, yellow; HS-5 in monoculture, dark green, in coculture, light green. x- and y- axes: UMAP1 and 2, respectively. **e** A small fraction of cells upregulates NF-κB-driven gene transcription. UMAP functional clustering based on the most abundant transcripts identifies seven transcriptionally homogeneous clusters (from 0 to 6, color-coded as indicated). The genes characterizing each cluster are reported in Supplementary Table 2 (cluster_markers). **f** NF-κB driven transcription is activated in both MM.1S and HS-5 cells upon coculture. GSEA analyses identify in MM.1S and HS-5 cells (left and right, respectively) some of the NF-κB driven pathways that are upregulated by coculture. False Discovery rate (FDR) is reported in each diagram.

Supernatants from static cultures of untreated MM.1S cells contained detectable levels of inflammatory cytokines and chemokines (CKs, Fig. 4a, left panel, grey bars) suggesting a link between the bona fide genetic NF-κB signature [9, 40, 41] and the non-zero NF-κB basal activation state (see Fig. 2c, d**)**. Although TNF-α strongly activates NF-κB in MM.1S cells, it did not induce significant changes in the overall CK content (Fig. 4a, red bars; Supplementary Fig. 11 a, b). This result might have at least a twofold interpretation: either CK changes are below the threshold of detection, or CKs are secreted only by the minority of hyper-responsive MM.1S cells leading to an undetectable CK increase in a two-hour stimulation. In the latter case, neighbouring cells might be activated and triggering a delayed response at population level.

Unstimulated HS-5 cells secrete almost undetectable levels of most NF-κB-activating CKs, apart from IL-6, IL-8 and CXCL5 (Fig. 4b), which is compatible with the minimal basal NF-κB activation. After 2 h TNF-α treatment, the secretory profile changed and some CKs increased two- to fourfold (DKK-1, G-CSF, GM-CSF, VEGF, Thrombospondin, MIF) or slightly decreased (IL-6, CXCL5, SerpinE1). In the supernatants of MM.1S cells, neither IL-1β nor IL-1α were detected, in contrast with the presence of a minimal amount of IL-1β protein in the supernatant of HS-5 cells, which correlates with IL-1β transcripts in HS-5 cells in mono- and coculture (see below and in Supplementary Fig. 11g, i).

Since myeloma and stromal cells share the same ME niches in the BM [27, 42], cell type-specific CKs secretion might reciprocally affect NF-κB activation. We used MM.1S-HS-5 mixed cultures as the simplest 2D model for the BM niche (Fig. 4c). Indeed, in MM.1S the basal NF-κB activation increases when in contact to HS-5 cells, either because of local CKs secretion or physical contacts with HS-5 cells (or both). In contrast, basal NF-κB levels in HS-5 cells were not perturbed by the presence of MM.1S cells (Fig. 4c).

To understand whether NF-κB activation by MM.1S-HS-5 crosstalk impacts on their transcriptional phenotype, we performed single-cell RNA-seq in both mono- and mixed cultures. The UMAP (Uniform Manifold Approximation and Projection for dimension reduction) plot clustered the cells in 4 populations (Fig. 4d). Coculture crosstalk for four hours rewires the MM.1S transcriptional outputs, as suggested by the separation between the orange and yellow clusters in the UMAP; no change is detectable for the HS-5 clusters in these conditions (dark and light green clusters). This result is in good agreement with the co-culture NF-κB activation in MM.1S cells seen by live imaging. A graph-based clustering approach based on homogeneous transcriptional signatures further functionally splits the clusters in seven subclusters (Fig. 4e). GSEA analyses highlighted the overall activation of “TNF-α signalling via NF-κB” and “Interleukin signalling” in MM.1S cells, and non-canonical NF-κB activation in HS-5 cells (Fig. 4f).

The same applies to the small Cluster 6 (lime green dots) that accounts for approximately 1% of the mixed populations (including both MM.1S and HS-5 cells), and expresses high levels of NF-κB driven genes. This suggests that cocultures has the potential to activate NF-κB driven transcription in highly responsive cells (Fig. 4e, Supplementary Fig. 11h, k).

Overall, our 2D in vitro model for molecular crosstalk reveals a small but significant p65 activation in MM.1S cells in short-term co-cultures that is reflected in NF-κB driven transcriptional changes.

#### Reciprocal myeloma-stroma crosstalk produces low-level NF-κB activation in a compact 3D ME

**Fig. 5:**
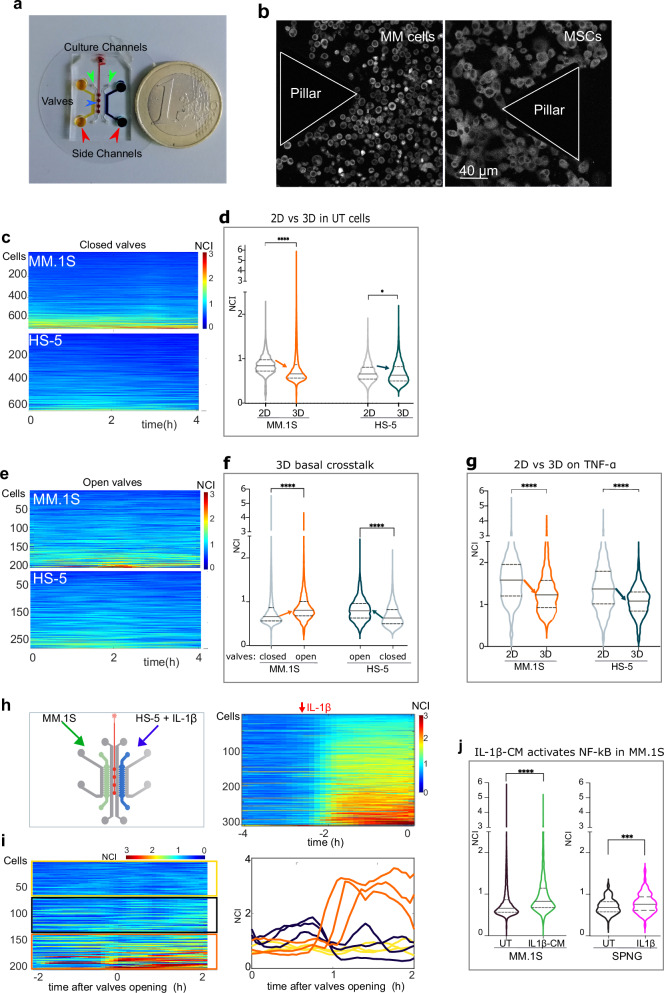
Stroma-MM bi-directional paracrine crosstalk in compact 3D microenvironments. **a** Custom microbioreactor size. The picture shows the device with cell culture chambers (orange and blue, red arrows) and doormat valves (dark red circles, blue arrow) connected by the red pressure line. In grey, cell loading and medium delivery channels (green arrows). MM.1S and HS-5 cells are plated in the yellow and blue chamber, respectively. One euro coin as scalebar. **b** Details of the microchambers with 3D plated cells. Confocal images of p65-YFP MM.1S cells (left panel) and p65-YFP HS-5 (right panel) plated in 3D microbioreactor chambers. Triangular structures (pillars) separate the culture chamber from the delivery channels (see also Supplementary Fig. 12 for technical details). Scale bar, 40 µm. **c** Dampening of basal NF-κB activity by compact 3D scaffold in both MM.1S and HS-5 cells, at a glance. Colorplots show NCI dynamics when MM.1S and HS-5 cells are cultured in separated chambers (closed valves). Representative experiment of the three performed. NCI values colorbar from 0 (blue, cytoplasmic) to 3 (red, nuclear). x-axis, time in hours. **d** A compact 3D scaffold dampens the basal NF-κB activity in MM.1S and HS-5 cells. Violin plots compare NCI values distributions for MM.1S and HS-5 plated in 2D cultures (grey, from Fig. 2) with those from 3D cultures in panel (**c**) (MM.1S: orange; HS-5: dark green). Data from one representative experiment of three performed. Arrows point to median values decreases. Statistical significance by ANOVA + Tukey’s multiple comparisons test (*P = 0.017, ****P < 0.0001). N° of cells and Median values are reported in Supplementary Table 3. **e** The reciprocal MM.1S-stromal crosstalk activates NF-κB dynamics in compact 3D scaffolds. Colorplots show NCI dynamics when MM.1S and HS-5 cells are cultured in chambers connected by open valves. Representative experiment of the three performed. NCI values colorbar, from 0 (blue, cytoplasmic) to 3 (red, nuclear). X-axis, time in hours. **f** A crowded 3D scaffold highlights NF-κB activation due to the reciprocal MM.1S-stromal crosstalk Violin plots compare NCI value distributions for MM.1S and HS-5 plated in separated 3D chambers in microbioreactors (grey, from panel **c**) and after valves opening and medium mixing (crosstalk, orange and green violins; colorplots not shown). Arrows indicate the median value increase due to crosstalk. Data from one representative experiment of three performed. Statistical significance by ANOVA + Tukey’s multiple comparisons test (****P < 0.0001). N° of cells and Median values are reported in Supplementary Table 3. **g** TNF-α driven NF-κB activation is dampened in 3D compact scaffolds. Violin plots compares p65 NCI distributions obtained from MM.1S and HS-5 cells stimulated with TNF-α in 2D cultures (in grey, from Figs. 2) or 3D microbioreactors (in colours). Representative experiment of the three performed. Statistical significance by ANOVA + Tukey’s multiple comparisons test (****P < 0.0001). N° of cells and Median values are reported in Supplementary Table 3. **h** IL-1β triggers NF-κB activation in MSCs. The cartoon shows cell loading geometry in the microbioreactor (green and blue chambers, respectively). IL-1β was added only to the HS-5-containing chamber (blue). The colorplot on the right shows NF-κB NCI in HS-5 for 2 h before IL-1β addition (−4 to −2) and further 2 h of stimulation before valves opening (−2 to 0). **i** IL-1β triggers a potent paracrine signal from stromal cells which activates NF-κB in 30% of MM.1S cells. The NCI colorplot shows NF-κB activity in MM.1S cells before (−2 to 0) and after valves opening when the IL-1β conditioned medium (IL-1β-CM) from HS-5 reaches MM.1S cells (0 to 2 h). Cells are ranked based on AUC. Yellow, grey, and orange boxes point to three MM.1S subpopulations with low, medium, and high activation, respectively. A selection of NCI dynamics from three representative cells from each subpopulation is shown on the right, with the same colour code. **j** IL-1β triggers a potent paracrine signal from HS-5 activating NF-kB in a fraction of the exposed MM.1S population. Violin plots compare NCI distributions from the experiment in panel **i** in untreated MM.1S cells before (grey, 2 h), and after (orange, 2 h) CM sharing. The two violin plots on the right compare NCI distributions for MM.1S cells in coculture with HS-5 on SPNG in the RCCS Bioreactor (UT, 24 h) and after 5 hr IL-1β treatment. Statistical significance by two-tailed non-parametric *t*-test (Mann–Whitney, *P* < 0.0001: ****P < 0.001: ***). N° of cells and Median values are reported in Supplementary Table 3.

Despite only partially reproducing the BM ME, which is rich in different cell types, vessels, fluids and 3D bone structures, microbioreactors represent reproducible experimental models for 3D cultures [43, 44]. Thus, we devised custom disposable microbioreactors suitable for 3D cell cultures on fibrin-based scaffolds. These tiny culture chambers mimic the crowded BM niche where cell-to-cell and cell-to scaffold contacts are frequent and secreted CKs do not dilute out (Fig. 5a, Supplementary Fig. 12 and Supplementary information relative to Fig. 5). In the microbioreactor, p65-YFP MM.1S and p65-YFP HS-5 cells were grown in microchambers physically separated by closed valves (Fig. 5b). NF-κB dynamics were analysed by live imaging as described above. Despite the absence of any flow, NF-κB basal levels in both HS-5 and MM.1S cells were very low, even lower than those in 2D-static cultures reported in Fig. 2a, suggesting that crowding and the 3D culture exert a dampening effect on NF-κB activation (Fig. 5c, d).

Valve opening allowed the reciprocal mixing of culture media bathing unstimulated HS-5 and MM.1S cells and led to a significant increase in NF-κB basal activation in both cell types (Fig. 5e, f), indicative of a reciprocal crosstalk. Data highlighted a paracrine signalling from MM.1S to HS-5 cells, previously undetected in 2D static cultures. Of note, a compact setup dampens also TNFα-driven NF-κB activation by 20% in HS-5 and MM.1S cells compared to 2D cultures (Fig. 5g).

The further logical step was to define the mechanism of paracrine crosstalk induced by inflammatory stimuli in 3D compact setups. TNF-α was excluded because it activates both cell types. However, IL-1β might represent the stimulus that activates NF-κB in HS-5 but not MM.1S cells (Supplementary Fig. 12. Moreover, IL-1β in our hands induces a secretory profile similar to that induced by TNFα (G-CSF, GM-CSF, DKK1, CXCL8, BSG), but with differences: MCP1, SerpinE1, CXCL1, CCL20 were higher in cells treated with IL-1β conditioned medium (CM, Supplementary Fig. 11e–g).

HS-5 and MM.1S cells were plated in separated microchambers (valves closed) and IL-1β added only to the HS-5 chamber. A two-hour incubation activated NF-κB in HS-5 (Fig. 5h, right). Valve opening let the IL-1β-CM to freely diffuse toward the myeloma cells. Time-lapse quantifications showed a substantial increase of p65 NCI in 25-30% of MM.1S cells (Fig. 5i, j**;** one representative experiment out of three performed). Responding cells were positioned all over the chamber and did not cluster close to the medium efflux ports (Supplementary Fig. 13a–d). Overall, a large fraction of myeloma cells in the population responded to IL-1β CM, including a substantial fraction of hyper-responders. A similar NF-κB activation in MM cells was reproduced by MM.1S-HS-5 cocultures on Spongostan in a RCCS bioreactor upon IL-1β stimulation (Fig. 5j).

Overall, our data showed that a crowded 3D microenvironment i) makes visible the paracrine bi-directional crosstalk between HS-5 stromal and MM.1S myeloma cells, ii) suggests that cell-to scaffold interactions exert a negative effect on the amplitude of NF-κB responses in both myeloma and stromal cells; iii) highlights that about 30% of MM.1S myeloma cells are extremely sensitive to molecules secreted by IL-1β-activated HS-5 stromal cells, iv) suggests that IL-1β is a key mediator in the crosstalk between HS-5 and MM.1S cells in our MM models, and potentially in MM patients.

#### IL-1β and paracrine signalling shape the myeloma BM ME

**Fig. 6:**
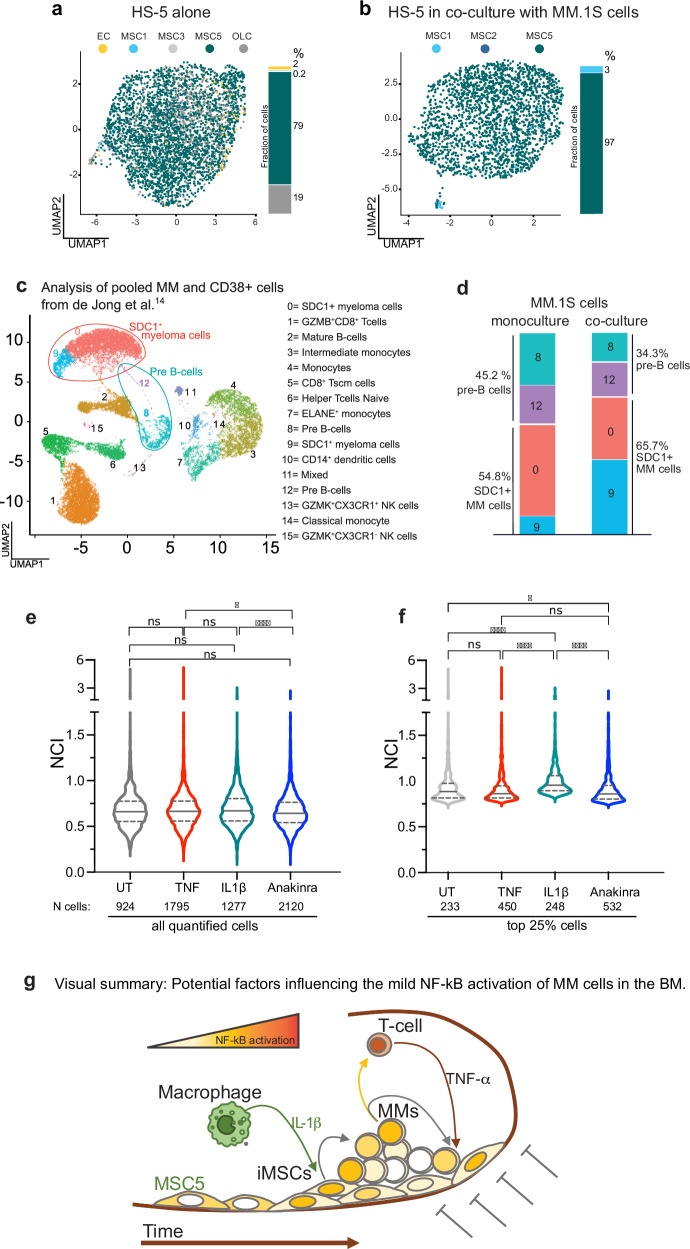
Soluble crosstalk in the BM niche modulates the transcriptional profiles in both myeloma and stromal cells. **a** HS-5 have a transcriptional phenotype similar to non-inflammatory mesenchymal stromal cells. UMAP data representation from our scRNA seq assigns identities to HS-5 in monoculture using the nomenclature reported in de Jong et al. [14] and colour coded as indicated in the legend: MSC 1 and 2 (inflammatory, myeloma specific, iMSCs, MSC1: light blue. MSC2 not present), MSC3 and 5 (MSCs present in healthy subjects, light grey and teal). OLC: Osteolineage cells (dark grey): EC: Endothelial Cells (yellow). The histogram on the right side of the UMAP plot report the frequency of each subpopulation as percentage. **b** A fraction of HS-5 acquires an iMSCs transcriptional phenotype when in coculture with MM.1S cells. Similar to panel **a**, UMAP representation assigns identities to single HS-5 cells upon coculture with MM.1S cells using the nomenclature reported in de Jong et al. for myeloma patients [14] and colour coded as indicated in the legend, MSC 1 and 2 (inflammatory, myeloma specific, iMSCs, light and dark blue), MSC5 (MSCs present in healthy subjects, teal). See also Supplementary Fig. 11 for population identification. The histogram on the right side of the UMAP plot reports the frequency of each subpopulation expressed as percentage. Cell numbers and frequency for each subpopulation are reported in Supplementary Table 3. **c** primary MM cells account for a large fraction of the immune CD38+ cells in the BM of myeloma patients. Extended data 6 from de Jong et al. [14] has been reanalysed by merging the subset of primary CD38+ immune cells with CD38 + /SDC1+ myeloma cells. All the cells are plotted in a single UMAP representation using the nomenclature reported in [14] and colour coded as indicated in the legend on the right. **d** The autocrine/paracrine signalling in MM.1S-HS-5 cocultures activates in MM.1S transcription profiles typical of myeloma cells. The transcriptional signature of MM.1S cells in monoculture (our sc-RNA seq in Fig. 4) identifies four types of cells which overlap with clusters 8 and 12 (pre-B cells) and clusters 0 and 9 (SDC1 + MM cells) identified in panel **c**. Similarly, the histogram on the right quantifies the changes in the subpopulation fractions in MM.1S cells before and after exposure to HS-5 for 2 hours. Population fractions are expressed as percentage (side of the histogram). Cell number and frequency for each subpopulation are reported in Supplementary Table 3. **e** IL-1β treatment recapitulates NF-κB activation in the mouse model. Violin plots compare NF-κB activation (NCI) in all the MM.1S cells quantified for p65 NCI in the calvaria of mice, which have been left untreated (UT, grey) or treated for 3 hours with TNF-α (red) or IL-1β (teal), or with Anakinra for 24 h (blue, IL1Ra IL-1β inhibitor). Number of cells analysed are reported below the x-axis. **f** IL-1β treatment increases NF-κB activation in a fraction of MM cells in the mouse model. The plot shows NCI value distribution for cells with high NCI (top 25%). Statistical analyses by ANOVA with Dunn multiple comparison test: significance as indicated in the plot: **** *p* < 0.0001, * *p* = 0.037, ns: not significant). **g** Visual summary: Potential factors influencing the mild NF-kB activation of MM cells in the BM. The schematic integrates our results for the reciprocal feedforward control on NF-κB activity in the BM niche of myeloma patients. In the inflammatory BM of myeloma patients, macrophages contribute to increase IL-1β levels which activates NF-κB-driven transcription in stromal cells (MSCs). Activated MSCs produce paracrine signals, which are captured by a small fraction of hypersensitive MM.1S cells. In turn, activated MM.1S cells both self-reinforce positive NF-κB feedback loops and send paracrine signals to neighbouring myeloma cells. Potentially, CKs secreted by MM.1S cells could stimulate T lymphocytes to deliver NF-κB-activating TNF-α to neighbouring MSCs and MM cells. The inflammatory ME might change the transcriptional phenotype of MSCs toward a more inflammatory one (iMSCs). In addition, being at the interface between myeloma cells and the bone, MSCs receive NF-κB dampening signals from the bone scaffold as happens in vitro, which counterbalance the inflammatory signals from the cellular niche. As a result, the in vivo ME is kept mildly inflammatory toward myeloma cells.

A recent paper [14] described inflammatory MSCs (iMSCs) as the predominant population in myeloma patients’ BMs. In the proposed model, cytokines IL-1β and TNF-α are likely produced by macrophages and T lymphocytes and are predicted to activate the inflammatory gene signature in MSCs and possibly drive the transition to iMSCs. To understand whether MM-secreted molecules, beside TNF-α and IL-1β, can impose the switch to iMSCs, we compared our scRNA seq data from four-hour mono- and co-cultures of HS-5 and MM.1S cells with those from primary MSCs in Ref. [14]. We found that 79% of our HS-5 cells in monoculture (from Fig. 4d) displays the physiologic MSC5 non-inflammatory stromal transcriptional landscape. The remaining cells are similar to iMSCs (MSC1/2, 0.2%), OLC (Osteolineage cells, 19%) and EC (endothelial cells, 2%) cells (Fig. 6a). Indeed, when HS-5 cells are exposed to myeloma cells for four hours, the iMSCs-like clusters increased roughly 10-fold to 2.3% (MSC1/2, Fig. 6b). The number of iMSC-like HS-5 cells is rather low, but this might be related to the very short exposure time in our experimental conditions as compared to the years-long disease progression.

We also re-analysed the scRNA seq data [14] from the primary CD38^+^ immune cell population upon merging with the myeloma population (Supplementary Fig. 6 in Ref. [14], Fig. 6c). Transcriptional features of monocultured MM.1S cells in our scRNA seq show a 55% overlap with the CD38^+^SDC1^+^ 0 + 9 clusters and a 45% overlap with the pre-B cell 8 + 12 clusters (Fig. 6d). Upon exposure to stromal cells, the CD38^+^SDC1^+^ myeloma-like clusters enlarged (69%) and the pre-B cell clusters shrank (31%), suggesting that a 4-hour stromal-to-myeloma crosstalk could be sufficient to prime transcriptional changes from the pre-B transcriptional landscape toward the more malignant CD38^+^SDC1^+^ one (Fig. 6d).

To confirm in vivo that IL-1β can be one of the key –although indirect– drivers of NF-κB activation in myeloma cells, 4 groups of 5 mice each were engrafted with MM.1S cells as described previously and left either untreated or treated for 3 h with TNF-α or IL-1β, or treated with Anakinra (IL1ra), a selective IL-1β inhibitor, for 24 h. Calvaria and femur bones were sectioned, stained for p65 and CD138 and imaged. NCI distributions in the four groups of mice were rather similar, excepted the significant (*p* < 0.0001) difference between IL-1β vs Anakinra groups (Fig. 6e and Supplementary Fig. 14a–c). When the NCI distributions in the top 25% of the populations were compared, differences become significant, suggesting that IL-1β, but not TNF-α, indirectly activates NF-κB in MM.1S cells populating the top 25% in the distribution. The inhibition of IL-1β signalling by Anakinra decreases the activation in the small fraction of naturally activated MM.1S cells in the BM (Fig. 6f and Supplementary Fig. 14c).

Hence, our data obtained in compact 3D microenvironments show that MM1.S exposure to molecules secreted by HS-5 cells exposed to IL-1β reproduces the NF-κB activation in myeloma cells seen in mouse calvaria and patients’ BMs.

Overall, our results indicate that IL-1β, acting on stromal cells, can be a key factor driving the MM-MSCs crosstalk in vivo. Furthermore, they suggest that the IL-1β inhibitor Anakinra can reduce the overall NF-κB activation in MM in the BM.

## Discussion

To get insights on NF-κB activation in myeloma and stromal cells at the micro- and macro-timescale, we set up 3D culture models building on our previous work on live single cell imaging and microfluidic devices. Our models concur in providing a more nuanced perspective of the traditional view of NF-κB as highly active in myeloma. They also highlight a soluble crosstalk between a fraction of stromal cells and a fraction of MM cells, triggered by IL-1β, a molecule with elevated levels in the blood of MM patients [4, 5].

Indeed, the IL-1β antagonist (IL-1ra) Anakinra has been used to delay or prevent the progression in patients with indolent or smouldering multiple myeloma [45, 46]. Preliminary results were not striking but a systematic evaluation is still missing. At present, Anakinra is widely used to control cytokine release syndromes, a dominant toxicity of anti-myeloma CAR T cell therapy, mediated in part by IL-1 [47]. Our results point to an IL-1β contribution in MM progression and encourage to refine the therapeutic protocols.

However, the intrinsic limitation of our approach consists in the use of a single myeloma cell line, MM.1S; despite our efforts, many other MM cells lines are not adaptable to culture models and cannot easily be genetically modified.

### Dysregulated NF-κB activity in MM cells

We used live cell imaging to characterize NF-κB activation at single cell level. We used MM.1S cells as a representative in vitro model due to their reproducible responses to inflammatory stimuli as opposed to MM cell lines with heterogeneous genetic lesions. Untreated MM.1S cells show fluctuating non-zero NF-κB activation levels on the timescale of minutes, and strong responses to inflammatory stimuli like TNF-α on the timescale of hours. Their inability to resolve NF-κB activation for many hours is indicative of defective feedback loops, a cell-autonomous feature. In addition, almost all the MM.1S cells in the population are primed to hyper-respond in vitro.

### NF-κB heterogeneity in the BM

A large fraction of MM cells in myeloma patients’ BMs show activated p65/NF-κB, but the activation is mild and heterogeneous. Such diversity in activation can have two non-mutually exclusive explanations: clonal genetic heterogeneity of MM cells, or low-level long-term activation of p65/NF-κB due to spatially restricted autocrine/paracrine secretion of NF-κB activating molecules. Recent studies documented the genetic heterogeneity of circulating primary MM cells, which partially reproduces the heterogeneity in the BM [48–50], suggesting that there might be a selective retention of MM cells in the BM. In contrast, the autocrine/paracrine hypothesis has never been addressed in living cells.

While our models do not reproduce clonal heterogeneity, we did find that MM.1S cells and –rather unexpectedly– also stromal HS-5 cells respond to stimuli with different kinetics in quasi-static environments, as opposed to environments with high exchange rate, suggesting that the autocrine/paracrine modulation of NF-κB activation can be microenvironment-driven.

Both MM.1S and HS-5 cells show less NF-κB activation in culture models resembling the BM. The nature of the molecules dampening NF-κB responses still needs to be investigated. In any case, a fast-exchanging medium –comparable to vessel transition zones in the BM [51–53]– enhances the intensity of NF-κB responses by removing secreted molecules possibly endowed with quenching activities. On the other hand, the build-up of paracrine signals, as might occur in recessed BM locations [51–53], might restrain NF-κB activity. Thus, the blood flow might control the spatiotemporal activation of NF-κB responses by dosing autocrine/paracrine signal concentrations around MM cells.

The protective effect of the niche conflicts with the inflammatory NF-κB signature found in circulating MM cells [54]. We speculate that circulating MM cells are exposed to high concentrations of NF-κB activating cytokines in the absence of any autocrine dampening.

### The crosstalk between stromal and MM cells

All our models indicate that stromal and MM cells crosstalk at the level of NF-κB activation. Stromal HS-5 cells appear to be more sensitive to NF-κB activating cues and to produce in turn soluble molecules that activate MM.1S cells. MM1S and HS-5 cells are both sensitive to paracrine TNF-α which cannot thus be the trigger of the crosstalk. However, high IL-1β levels are detected in patients’ blood [14] even after remission [4, 5] and several IL-1β gene polymorphisms are associated with worse prognosis [55]. When provided to our in vitro and in vivo MM models, IL-1β produces a strong direct NF-κB activation in HS-5 cells with a consequent indirect activation of MM.1S cells. However, it is worth mentioning that also IL-1α is expressed by HS-5 cells and can have an additive effect with IL-1β to NF-κB activation. In our mouse MM model, the treatment with the IL-1 receptor antagonist Anakinra dampened the NF-κB activation, especially in the most responsive cells.

The importance of the non-malignant ME in cancer pathobiology is being increasingly acknowledged and local, often low-grade, subclinical inflammation is considered an important driver of tumour development, growth and resistance to therapy in various malignancies [56]. Recently, the Sonnenberg and Cupedo labs [14] put forward the hypothesis that a large fraction of inflammatory MSCs (iMSCs) would be instrumental to prime the BM for myeloma proliferation and immune cell modulation. Our models support their hypothesis that the in vivo MM-MSC crosstalk reciprocally influences the transcriptional output.

The potential source of IL-1β in the TME was not investigated. However, macrophages [57–59] and frequently MM cells themselves [60] are possible contributors. In addition, an IL-1β-driven crosstalk between neutrophils and MM cells in the BM has been recently described [61].

In summary, our data indicate that i) NF-κB activation in the BM is mild and heterogeneous, ii) is due to the activation of a minority of cells, iii) is dampened by restraining physical microenvironmental cues and iv) can be triggered by IL-1β-dependent stroma-to-MM crosstalk and can be reduced by treatment with Anakinra.

The graphic summary in Fig. 6g describes how our findings can be integrated in a simple representation of the BM microenvironment: a soluble crosstalk driven, at least partly, by IL-1β from monocytes/macrophages [62] and TNF-α from T lymphocytes [63] in the cellular niche activates NF-κB either directly or indirectly in stromal and myeloma cells. In contrast, dampening signals originating by interactions with the bone matrix, possibly through cell-adhesion activated mechanisms, counteract inflammation in the ME [64].

## Materials and methods

### Study design

Our work aimed at understanding how the interactions between cell-autonomous and cell-extrinsic factors shape the NF-κB response in myeloma cells to inflammatory stimuli present in the BM microenvironment.

We recently demonstrated that live imaging of NF-κB oscillations in basal and perturbed conditions provides a deeper understanding of NF-κB regulated transcription in different time windows [16].

We therefore generated myeloma and mesenchymal stromal cells expressing physiological levels of p65-YFP proteins by knocking-in the YFP Open Reading Frame at the 3’end of the p65 gene, which have never been prepared before. We then applied high through-put live imaging, microfluidics and microbioreactors setups in combination with single cell RNA seq. We also quantified NF-κB localization in MM cells engrafted in the calvaria bones of mice exposed or not to TNF-α, IL-1β and its inhibitor Anakinra.

Our experimental approach has three arms: the first described with precision NF-κB localization in MM cells dwelling in the BM of mice and patients by high throughput quantitative imaging. The second arm took advantage of experimental devices mirroring different properties of the 3D microenvironment to quantify the contribution of cell autonomous and cell extrinsic components in modulating NF-κB activation. In the third arm, the effect of IL-1β in NF-κB activation has been verified in the mouse model.

### CRISPR/Cas9 engineering of the endogenous p65 gene (knock-in, KI)

The map for the knock-in of the Venus fluorescent protein coding sequence at the 3’end of the endogenous p65 locus is shown as Fig. 2a: RefSeq NC_000011.10 (65653601…65662946, complement); NM_021975.4; Ensembl: ENST00000406246.8; GeneID: 5970 (RELA); HGNC: 9955; MIM: 164014).

eFLUT (Endogenous Fluorescent Tagging Toolkit) plasmids were kindly provided by Prof. Galit Lahav (Harvard Medical School) [32].

MM.1S [25] and HS-5 [29] cell lines were transduced with a Cas9-expressing lentivirus (lentiCRISPR v2 plasmid #52961 by Addgene) and then nucleofected (4D-Nucleofector™ System by Lonza, kit Cat. no. V4XC-2032) with guide RNA (crRNA, tgagtcagatcagctccTAA), transactivating RNAs (tracrRNA, (Alt-R® CRISPR-Cas9 System, IDT), and donor DNA produced by PCR and containing the Venus Open Reading Frame (ORF) flanked by the two homology arms. Transfection protocol was designed and optimized as for manufacturer’s instruction. After 2 weeks of Hygromycin B selection (Sigma-Aldrich H0654, 50–100 μg/ml), the YFP-positive polyclonal cell populations were FACS sorted (Flow Cytometry, MoFlo XDP Cell Sorter), FACS analysed with a NAVIOS flow cytometer (Beckman Coulter), and data analysed using FCS Express 6 software (De Novo Software). The signal intensity of wildtype cells was set as background and the signal of YFP positive cells (514 nm laser, band pass filter 525/540) recorded. All the samples were analysed keeping parameters constant.

### Cell cultures

#### Cell lines and culture conditions

MM.1S (human multiple myeloma B lymphoblasts, https://web.expasy.org/cellosaurus/CVCL_8792; https://www.keatslab.org/myeloma-cell-lines/hmcl-public-availability) [25], JY **(**EBV-LCL) and HS-5 [29] (human bone marrow stromal cells) were purchased from the American Type Culture Collection (ATCC, Manassas, VA) Other human myeloma cell lines, U266 (U266B1 - TIB-196, ATCC), OPM2 (https://www.cellosaurus.org/CVCL_1625) and RPMI8226 (RPMI 8226 - CCL-155, ATCC) were a kind gift of Prof. Giovanni Tonon, San Raffaele Scientific Institute, Milan, Italy.

Normal B cells, from San Raffaele Biobank.

Myeloma and primary B cells were cultured in phenol-red free RPMI 1640 (RPMI, Gibco) while Dulbecco’s Modified Eagle Medium (DMEM, Gibco by ThermoFisher Scientific) was used for HS-5, with the addition in both of 10% v/v foetal bovine serum (FBS, Gibco), 1X L-glutamine (100X L-Glu, 25030149, Gibco), 1X Penicillin-Streptomycin (100X, #15140122, Gibco).

Maintenance: Cells were seeded at 5 × 10^5^ cell/ml 3 times/week, kept in culture for 1 month maximum in tissue culture plastic (Corning by Sigma Aldrich) and routinely tested for mycoplasma contamination by polymerase chain reaction (PCR).

#### Cell treatments

Cells were stimulated with cytokines or chemokines (CKs): TNF-α, IL-6 and IL-1β (10 ng/ml) or of SDF-1 (160 ng/ml). Stimuli were added to the medium delivered to either static chambers or to flow chambers in CellASIC plates or microbioreactors.

#### Co-cultures in 2D

p65-YFP MM.1S cells (5 × 10^4^) were added to a monolayer of wildtype (wt, dark) confluent HS-5 for p65-YFP quantification in MM.1S cells, or 5 × 10^4^ wtMM.1S cells (dark) on a monolayer of p65-YFP HS-5 for p65-YFP quantification in HS-5, 8-well chambers (Nunc™ Lab-Tek™; area 0.8 cm^2^) were Fibronectin-coated. Plates were incubated overnight in a cell culture incubator. The day after, live imaging acquired images as described in 3. “Live imaging and quantifications”.

#### Static and continuous flow culture set-ups for live imaging

*Static 2D cultures*: p65-YFP MM.1S and HS-5 cells were plated on Fibronectin-coated 8-well chambers (Nunc™ Lab-Tek™) at 5 or 2 × 10^4^ cells/well, respectively, 1 or 2 days in advance respectively. NucBlue dye (1:100 dilution, Invitrogen) was added to cells immediately before incubation on the microscope stage at 37 °C for 1 h to stabilize the plate.

Ten images (60 min) were recorded to quantify the starting basal level of each cell then TNF-α diluted in culture medium was gently added to the wells. Culture medium was added in mock untreated cells.

In a standard experiment, for each experimental point, two wells were imaged at 10 field/well every 6 min. Each experiment has been repeated 3 to 6 times unless otherwise stated in the Results section.

*Continuous 2D flow culture*: Cells were plated in commercial 4-chamber microfluidic plates (CellASIC® ONIX plate for mammalian cells, M04S-03-5PK by Merk Millipore, see also Supplementary Fig. 8) after Fibronectin bottomglass pre-coating (10 µg/ml, 1 h at RT). p65-YFP MM.1S cells or HS-5, 3 × 10^3^ or 1 × 10^3^ cells/chamber were plated 1 or 2 days in advance, respectively. The CellASIC® ONIX Microfluidic Platform (CellASIC® ONIX Microfluidic System Package, EV262, by EMD-Merk Millipore, KGaA) connected to the CellASIC® ONIX Tri-gas Mixer (GM230, by Merk Millipore) applies a continuous medium flow (rate: 0.5 psi, 10 µl/h, chamber volume of 1 µl). The vital nuclear dye Hoechst 33342 (HOE, 1:100 v/v NucBlue, Invitrogen) was added in all the media, TNF-α in selected chambers for 6 to 10 h as described. To refresh the overnight culture medium, deliver HOE and remove secreted molecules and cell debris, the plate was pre-incubated under flow for 90 min on the microscope stage. Afterwards, a flow at 0.5 psi for 720 min imposed from channel 2 + 4 and 3 + 5 symmetrically delivered the medium ± TNF-α s to the cells in the culture chambers (Supplementary Fig. 8).

*Comparison of cell numbers per area or per volume*: Data comparing cell concentrations in the four different cultures per surface or per volume unit are reported in Supplementary Table 3.

### MM.1S-HS-5 cocultures in the Rotary Cell Culture System (RCCS) Bioreactor

Scaffolds were seeded in a bioreactor as described [28]. Briefly, p65-YFP MM.1S cells (5×10^5^/scaffold) and dark HS-5 cells (2.5 × 10^5^/scaffold) were seeded on six Spongostan standard^TM^ scaffolds (Ethicon, Johnson & Johnson, Belgium) following the timeline below.

Experimental timeline:

T0 (day1): HS-5 are added to hydrated Spongostan (SPNG) scaffolds in 96well low-adhesion plates.

T4h: scaffolds are transferred in dynamic culture vessels overnight (600 µl).

T 24 h (day2): MM.1S cells resuspended in 600 µl are added to the vessels and dynamic-cultured for 5 h.

T 29 h: medium volume is brought up to 10 ml/vessel with 1:1 dilution of DMEM and RPMI + 10% FCS and grown undisturbed for 24 h in dynamic cultures.

T29 to T53h: Incubation before scaffold withdrawals as reported in Fig. 1d. Scaffolds were either in vivo imaged or PFA fixed, paraffin embedded (FFPE) and sections for IF/IHC analyses prepared with a LeicaBiosystem station (EG1150H and RM2235). Each experiment has been repeated twice. Quantification of nuclear p65 was calculated as described in “Matlab software for NCI quantification” section and expressed as NCI.

### Human bone marrow samples

Human BM PPFE sections from myeloma patients were obtained from the Pathology Department (Ospedale San Raffaele, Milan, Italy) (approved by the Ethical Committee of the San Raffaele Institute). Male and female patients were treatment-naïve and heterogenous per age. BM biopsies at diagnosis presented different degree of plasmacells infiltration and cellularity as follows:

Pt.1: age 58, male, infiltration 30%

Pt.2: age 74, female, infiltration 80%

Pt.3: age 82, male, infiltration 60%

Pt.4: age 70, male, infiltration 50%

Pt.5: age 67, female, infiltration 90%

Pt.6: age 71, female, infiltration 50%

Pt.7: age 71, female, infiltration 80%

Pt.8: age 57, male, infiltration 50%

Pt.9: age 80, male, infiltration 60%

### Live imaging and quantifications

#### Live imaging to record p65 dynamics

Live cell imaging was performed on a TCS SP5 confocal microscope equipped with a 37 ˚C, 5% CO_2_, humidified chamber. Nuclei stained with Hoechst 33342 (HOE, 1:1000 dil, NucBlue™ Live ReadyProbes™ Reagent, ThermoFisher) were imaged with 405 nm UV laser (HOE channel), while low-intensity Argon laser 514 nm captured p65-YFP fluorescence (YFP channel). A dry 20X objective (0.7 NA) with 2X electronic zoom was used to obtain 1024×1024 pixel images with 16-bit pixel depth. The pinhole was set at Airy3 to capture the fluorescence coming from the whole cell thickness (approx. 10 µm) with timelapse intervals of 6 minutes up to 12 hours [16].

Proprietary format Leica.lif files containing nuclei and p65 signals were exported as 16-bit TIFF files. Nuclei were segmented and YFP intensity quantified as nuclear/cytoplasmic intensity (NCI) using a custom-made MATLAB® code (see NCI quantification below).

To assess whether localization dynamics of endogenous p65 and YFP-tagged p65 are comparable, NCI value distributions were normalized (Z-score normalization, Z = (x-mean) / (standard deviation)).

Every live imaging experimental condition was acquired in two technical duplicates and each experiment was repeated two to four times, as indicated in the figure legends. Statistical analyses are described below.

#### Matlab software for NCI quantification

Segmentation, tracking of cell nuclei and calculation of p65 Nuclear-to-Cytoplasmic Intensity (NCI) were performed via custom-made MATLAB® code adapted from ^2316^ with minor modifications to capture the intensity of the thin cytoplasm for MM cells.

For SPNG sections, and patients’ and mice BM samples, we used CellPose [65] for a more accurate nuclear and cytosolic segmentation, from which NCI was calculated as previously described.

#### NCI representations and p65 quantitative descriptors

Colorplots visualize p65 dynamics in each cell, according to an NCI colormap ranging from 0 (blue, total cytosolic localization) to 3 (red, strong nuclear localization). We also propose the use of additional intensity descriptors besides NCI (cartoon in Fig. 2e). More specifically: maximal value of activation (NCI_max_) and the time-integrated activity of nuclear p65 or Area Under the Curve (AUC). The most informative time descriptors considered have been the lag-time to reach the maximal response (Time-to-NCI_max_, T_max_ for short), the total time in the activated state (T_act_, summation of the time intervals with NCI values over the activation threshold of NCI = 1.2 for both MM.1S and HS-5) and T_decay_ as the time spent by the NCI curve to decrease below the activation threshold.

### Reagents

8% PFA, Electron Microscopy Sciences, 157-8

NF-κB p65, rabbit monoclonal (D14E12) XP® #8242, Cell Signaling

NF-κB p65 mouse monoclonal (33-9900, Invitrogen)

CD138, mouse monoclonal Ab, #356502 Biolegend,

CD44 mouse monoclonal Ab, NB100-65905, NovusBio

β -tubulin, mouse monoclonal clone SAP.4G5, T7816, Sigma Aldrich

β -actin, mouse monoclonal, clone AC15, A5441 Sigma Aldrich

GAPDH, mouse monoclonal clone GAPDH-71.1, G8795, Sigma Aldrich

Goat-α−Rabbit, -α−Rat, -α−Mouse secondary fluorescently tagged AlexaFluor antibodies (Molecular Probes, Invitrogen)

Normal Goat Serum (566380 Sigma-Aldrich)

Fibronectin 10 µg/ml (Roche Diagnostics, #10838039001)

Spongostan standard^TM^ scaffolds (Ethicon, Johnson & Johnson, Belgium)

IL-6 (Peprotech, #200-06), 50 ng/ml

IL-1β (Peprotech, #200-01B), 10 ng/ml

CXCL12 (HMGBiotech Srl, Milan), 5 ng/ml

TNF-α (Tumor Necrosis Factor-α, Recombinant, R&D Systems, Cat N°P01375), 10 ng/ml

Hygromycin B for recombinants selection (Sigma-Aldrich H0654), 50–100 μg/ml

NucBlue™ Live ReadyProbes™ Reagent (Hoechst 33342) N°R37605, Invitrogen™, 1:1000 dilution

### Immunostaining

#### p65 immunostaining of cells

Cells plated on Fibronectin-coated coverslips in duplicates were fixed for 10 min at RT with 3.7% Paraformaldehyde in PHEM buffer (60 mM PIPES, 25 mM HEPES, 10 mM EGTA, 4 mM MgSO_4_, pH7), and permeabilized with ice-cold Sucrose-HEPES-Triton-X100 buffer, for 5 min on ice. Coverslips were washed, blocked in 4% BSA, 10% Normal Goat Serum and eventually incubated with anti p65 antibody (1:200, see reagent list) overnight at 4 °C. After washing, coverslips were incubated with the appropriate secondary antibodies, nuclei stained with NucBlue, and images sequentially acquired with a 20X, 0.7NA or 63×1.4NA objectives using Leica TCS SP5 confocal microscope equipped with Diode 405 nm (nuclei) and Argon 514 nm (p65-YFP fluorescence) lasers obtaining images at 1024×1024 pixel resolution with 16-bit pixel depth.

#### IF and IHC of human BM and scaffold sections

SPNG sections from Formalin Fixed Paraffin Embedded (FFPE) scaffolds and human BM sections from FFPE BM biopsies from myeloma patients from the Pathology Department (Ospedale San Raffaele, Milan, Italy, approved by the Ethical Committee of the San Raffaele Institute, BioBank 09052013) were dewaxed and slides underwent Citrate antigen retrieval and IHC staining in a BOND-X Fully Automated Staining System Instrument (Leica Biosystems, Nussloch GmbH 2021). Images of Haematoxylin&Eosin stainings were digitally acquired with the Leica “Aperio AT2” System equipped with a 40X obj (Leica, Nussloch GmbH 2021). Alternatively, after antigen retrieval, sections were manually stained by IF and imaged with the Leica TCS SP5 confocal microscope following a standardized protocol (described above). Antibodies for both manual and automatic staining are listed in the “Reagents” section above. Mouse calvaria bones staining is reported in the Mouse engraftment and calvaria bones staining section.

#### Total protein quantification by Western blot

Briefly, cells were lysed in 1X reducing Laemmli buffer (1 × 10^4^/µl of 2% SDS, 0.05% 2-mercaptoethanol, 10% Glycerol, 0.0005% bromophenol blue, 0.063 M Tris-HCl, pH 6.8) incubated at 95 °C for 10 min, loaded onto 10% PAGE gels. Proteins were separated at 80–120 V and transferred to a nitrocellulose membrane using the Trans-Blot® Turbo Transfer TM System, BioRad. Membranes blocked for 1 h at RT with 2% w/v ECL blocking agent (GE Healthcare UK Limited) in TBST 1X (Tris-Buffered Saline, 0.1% Tween 20) were stained with a rabbit mAb anti NF-κB p65 (1:500, D14E12, Cell Signaling). Signals for β-actin, beta tubulin or GAPDH proteins were used as internal normalizers.

Goat anti-rabbit or goat anti-mouse Cy5-conjugated (Molecular Probes) secondary Abs were used. Dried membranes were imaged with Typhoon Fluorimeter FLA 9000. Images (16-bit, 50 µm pixel size) were exported as.tif files. Band intensities were quantified with the Fiji software [66]. Two to three replicates were evaluated for normalized total intensity and fold-changes calculation between treated and untreated samples. Statistical significance was assessed using Student *t*-test in Excel Spreadsheet.

### Custom microbioreactor platforms, design, and manufacture

The microbioreactor was conceived with two microchambers for 3D cell culture and consists of two functional layers: (i) the culture chamber layer, and (ii) a pressure-actuated compartment to regulate doormat valves to open or close communication between the two cellular compartments (see Fig. 5 and Supplementary Fig. 12 for visual details). Manufacture was performed at the Polifab -the micro- and nanofabrication facility of Politecnico di Milano, Italy.

*The culture layer is composed by two symmetrical culture channels* (7 mm long, 1 mm wide from the pillar to the inner wall) delimited by 13 triangular posts (“pillars”, visible in Supplementary Figs. 12 and 13) and a single side channel (700 µm wide) connected to reservoirs for culture medium replacement and biochemical cell stimulation. The length of culture channel, dimensions of the pillars and surface tension were optimized to confine the hydrogel exclusively in the culture channels, while providing free flow of nutrients and chemicals to cells.

*The pressure actuated compartment* consists of an actuation line with 4 round Doormat valves in series [126] (14.5 mm length, 200 μm height, Ø 900 μm each).

*Device master moulds* for both the culture layer and the pressure actuated compartment were produced by conventional photolithography in a cleanroom (Class ISO 6) at Polifab (PoliFab, Politecnico di Milano). Drawings of the two layers realized by CAD software (AutoCAD, Autodesk Inc.) were photomasked by a laser printer (MicroLithography Services Ltd., Essex, UK). Geometric patterns were printed at full size, high-resolution (64000 dpi) on Mylar® polyester film. The pattern of each layer was transferred on SU8-2100 photoresist (MicroChem, USA), previously spincoated on 4” polished silicon wafers. A final thickness of 200 μm was achieved by the superimposition of two 100 μm layers carefully aligned through a mask aligner (Carl Süss, mask aligner). Specifically, to obtain thickness of 100 μm the spinning rate was set to 3500 rpm. Adequate alignment was checked by an optical transmitted light microscope (INM 200, Leica) and the overall layer thickness measured through a profiler (KLA Tencor P-15 Profiler).

*Microbioreactors* were realized in polydimethylsiloxane (PDMS, gas permeable, biocompatible, and optically transparent) mixed with the curing agent (10:1 w/w, Sylgard 184, Dow Corning); the degassed mixture was casted on the two master moulds. After a 2 h curing at 65 °C, the pneumatic layer was peeled off and an access port punched (1.5 mm biopsy puncher). This layer was carefully aligned and permanently bonded onto the culture layer by 50 s of air plasma treatment (Harrick Plasma Inc.) and 30 min at 65 °C. The complete device was peeled off from the culture layer master mould and holes for media reservoir and ports for hydrogel injection were made with 3 mm and 750 μm biopsy punchers, respectively. The device was irreversibly bonded to a round borosilicate glass coverslip (Thermo Fisher Scientific, Ø 35 mm, 0.13–0.17 mm thickness) with 50 s of plasma treatment, taking care to actuate the valve layer to avoid irreversible bonding of the “corridor” between culture channels to the glass. The valves were kept open by a syringe-applied negative pressure through a Tygon tube clamped right after valves opening (TYGON®Saint-Gobain Performance Plastics, inner Ø 500 μm). The device was left at 65 °C overnight before use. An example of complete 2-layer microchamber CHIP device is illustrated in Supplementary Fig. 12.

*Microbioreactors validation*: The minimum negative vacuum pressure required to open and create connection between the two culture chamber channels was identified by applying a negative pressure (vacuum gauge, Tygon tube) to the actuation layer filled with a red color food dye (Mallard Ferrière). Pressures were tested in the 0 mmHg to −600 mm Hg range, with −100 mmHg steps. The effective valve opening was assessed in three independent microbioreactors. 8-bit gray scale images were acquired with transmission microscope equipped with a digital camera (AmScope Microscope Digital Camera MU500) at 4X magnification at each pressure step. Mean grey intensity on inverted images was evaluated with ImageJ software by manually defining a region of interest (ROI). Mean intensities of the ROIs were background subtracted.

*3D cell seeding inside the microchambers*: Devices are autoclave sterilized (Tanzo Classic WOSON Steam Sterilizer). Cells were resuspended at 2.5 × 10^5^ p65-YFP MM.1S or 2.0 × 10^5^ p65-YFP HS-5 in 10 μl of fibrin hydrogel, obtained by mixing 1:1 v/v of 10 mg/ml Fibrinogen and 5 U/ml of Thrombin. Cells were injected and the hydrogel was cross-linked at 37 °C for 5 min. After fibrin polymerization, complete medium containing 1:1000 v/v NucBlue (Invitrogen) was added to the culture medium in the side channels. Devices were left in the incubator overnight and imaged on the next day.

*Microchambers hydraulic independence.* Valves must warrant hydraulic independence of the two chambers. In addition, valves opening should provide fast molecules diffusion between the two culture chambers. To test these features, with closed pneumatic layer, one culture chamber was filled with a solution containing a small protein (0.5 μg/ml BoxA, HMGBiotech Srl, Milan) conjugated with the fluorescent dye ATTO 532 nm with MW 12 kDa, which is comparable with those of most cytokines and can mimic the diffusion of paracrine factors released by the cells (i.e., TNF-α 17 kDa). Independency of the chamber layer was monitored by keeping the valves closed for 2 h before opening them and enabling chambers communication. To evaluate diffusion time from one channel to the other, Atto532-BoxA fluorescence in the two chambers were immediately time-lapse imaged at 2 min/frame with low-intensity Argon laser confocal microscopy (514 nm, Leica TCS SP5). Device features were acquired in the optical transmission channel.

### Cytokines and chemokines quantification

MM.1S cells (3.5 × 10^5^/ml) and HS-5 (5 × 10^4^/cm^2^) were plated at day 0 and stimulated at day 1 with either medium or 10 ng/ml TNF-α. Supplementary Fig. 11 shows CK quantifications from HS-5 cells stimulated with 10 ng/ml of IL-1β. After 2 h incubation, supernatants were centrifuged at 4 °C at 15,000 *g* and stored at −80 °C. CKs were quantified with a membrane-based assay (Proteome Profiler Human XL Cytokine Array Kit, ARY022B, R&D Systems) with minor modifications. Briefly, nitrocellulose membranes spotted with antibodies to human CKs in duplicate, were incubated with supernatants at 4 °C for 16 h. Captured CKs were detected with provided biotinylated antibodies. Phycoerythrin-tagged Streptavidin replaced the provided Streptavidin-Peroxidase detection enzyme to collect the signals in a wider dynamic range with a fluorimeter (Typhoon FLA 9000, GE Healthcare, 488 nm laser, 16-bit depth tiff-images with 10 µm/pixel size resolution). Quantifications were performed for two independent experiments, two filters for each experimental condition (technical replicates) were acquired. Background-subtracted signal intensities were calculated with FiJI software [66]. To compare independent experiments, the fluorescence intensity of each spot was normalized to the internal reference controls. CKs fold change upon stimulation is obtained by dividing the normalized spot signal from treated supernatants by the spot signal from the untreated sample. Statistical significance of CK differences between CTRL and TNF-treated supernatants was determined by *t*-test without correction for multiple comparisons using GraphPad Prism software (GraphPad Software Inc.).

### Single cell RNA sequencing

*Single-cell RNA sequencing* Approximately 2 × 10^6^ MM.1S and HS-5 cells from monocultures and 2 × 10^6^ cells after four-hour MM.1S-HS-5 coculture were washed with PBS + 0,01% BSA, filtered with 40 μm strainer and resuspended to 200 cell/μl. *Single-cell RNA sequencing* was performed in collaboration with the Center for Omics Sciences (COSR) in San Raffaele Institute. Drop-seq [67] was applied on single cell suspensions using PDMS microfluidic device for encapsulation (Nanoshift, USA). Approximately 4,000 cells per experimental point have been sequenced at an average of 45 × 10^3^ reads/cell. *A*nalyses were performed using Seurat (v2.4, Butler et al. 2018, https://satijalab.org/seurat/archive/v2.4/pbmc3k_tutorial.html).

*Comparison of our datasets with* datasets from “The multiple myeloma microenvironment is defined by an inflammatory stromal cell landscape” paper [14].

Single cell RNA-seq datasets from mono- or co-culture of HS-5 and MM.1S cells produced in our lab and in-house analysed in Fig. 4d, e), were compared to single cell RNA-seq datasets from CD38^-^ nonhematopoietic or CD38^+^ hematopoietic cells, kindly provided by. Dr. Sonnenberg and Dr. Cupedo with the input count matrixes (https://github.com/MyelomaRotterdam/Microenvironment).

The De Jong datasets were re-analyzed by our bioinformatician with Standard Seurat protocols to obtain an inclusive map of all the hematopoietic CD38^+^ cells, and position also the myeloma cell clusters. After label transfer using the FindTransferAnchors and TransferData Seurat functions, the PCA structure of a reference object was projected onto a query object returning a predicted cell-type annotation which allowed to compare the transcriptional phenotypes in MM.1S in monoculture or in coculture with HS-5 cells with the hematopoietic clusters in De Jong et al.

The same FindTransferAnchors and TransferData Seurat functions have been used to compare the transcriptional profiles of MSCs in mono and coculture with those defining the 5 MSCs clusters spanning from MSC1-2 (inflammatory, myeloma specific) to MSC 4, 5 and 6 (present in both healthy donors and myeloma patients).

A list of cluster-defining genes is added as Supplementary Table 2, cluster markers.

Throughout figures, the cluster identities are represented with the same colour indexes to facilitate comparisons.

Our datasets available to Editors and Reviewers can be found at the GEO data repository: https://www.ncbi.nlm.nih.gov/geo/query/acc.cgi?acc=GSE197936 and accessible with the token wxybacukndejngr.

### Mouse transplantation and calvaria bones staining

NSG mice (females, 8–12 weeks, Charles River, Calco, CO, Italy) were used for experiment shown in Figs. 1 and 6. The experiment was repeated twice, with 5 to 7 animals for each experimental point as indicated in the text. All mice were housed under standard specific pathogen–free conditions and allowed access to food and water ad libitum. All efforts were made to minimize suffering. All the procedures on animals were conformed to Italian law (D. Lgs N°. 2014/26, implementation 2010/63/UE) and approved by the Italian Ministry of Health (authorization N° 127/2012-A, 04/06/2012, D.lgs. 116/92). All experimental protocols were approved by the San Raffaele Institutional Animal Care and Use Committee and by the Italian Ministry of Health (IACUC N° 1161 to AA).

Mice were i.v. injected with 10^6^ p65-YFP MM.1S cells in PBS and sacrificed after 20 days.

For experiments in Fig. 6, before sacrifice, mice were i.v. treated with 100 µg/kg IL-1β or 0.5 µg/mouse TNF-α for 3 h. Anakinra was injected 24 h before sacrifice at 25 mg/kg. Mock treated mice received PBS.

Femurs and calvaria bones from skulls were recovered from isofluorane euthanized mice, PFA fixed, OCT-included by standard procedures and stored at −80°. Cryostat sections were manually stained with Hoechst 33342 for nuclei visualization or immunostained for p65 as described. Confocal images (63x, 1.4 NA objective, Airy1, 16 bit, 1024×1024 pixels) were acquired with a Leica TCS SP5 confocal microscope at 405 and 514 nm for DNA and p65-YFP fluorescence, respectively. Brightfield images show the bone structures surrounding the cells. p65/NF-κB NCI was quantified as described in the “Matlab software for NCI quantification” section.

### Statistics

All data representations and statistical tests were performed using GraphPad Prism and MATLAB Software. Two-dataset comparisons were performed by non-parametric, unpaired, two-tailed Mann-Whitney *t*-test (compare ranks). Multiple comparison tests have been performed by ANOVA with Tukey’s or Bonferroni corrections or Kruskal-Wallis with Dunn’s correction, as indicated.

Statistical significance of CK differences between CTRL and TNF-α treated supernatants was determined by Kolmogorov-Smirnov *t*-test.

*P*-value significance >0.05 or <0.05, 0.01, 0.001, 0.0001 is represented in plots and text as ns, *, **, ***, ****, respectively.

Dynamic data distributions were mainly represented by violin plots; a solid line is the median value, while dotted lines indicate the 25% and 75% quartiles.

N° of analysed cells and Median values are reported in Figure legends.

## Supplementary information

Supplementary material

Supplementary table 1

Supplementary table 2

Supplementary table 3

Supplementary video 1

Supplementary video 2

## Data Availability

All data in the main text or the **Supplementary** materials are available upon request. Single-cell RNA sequencing datasets of MM.1S and HS-5 cells in mono or mixed cultures are available on GEO accession GSE197936. Go to https://www.ncbi.nlm.nih.gov/geo/query/acc.cgi?acc=GSE197936. Enter token wxybacukndejngr into the box.
