# Supplementary material for: In vitro models of the crosstalk between multiple myeloma and stromal cells recapitulate the mild NF-κB activation observed in vivo
Source: Cell Death Dis. 2024 Oct 6;15(10):731. doi: 10.1038/s41419-024-07038-1 (PMC11456592; doi:10.1038/s41419-024-07038-1)
Supplement: Supplementary file 1 — Supplementary material [file 41419_2024_7038_MOESM1_ESM.pdf]

Title:

In vitro models of the crosstalk between multiple myeloma and stromal cells recapitulate the mild NF-κB activation observed in vivo

Authors:

Federica Colombo[1,2§], Virginia Guzzeloni[3§], Cise Kizilirmak[1], Francesca Brambilla[1], Jose Manuel Garcia-Manteiga[4], Anna Sofia Tascini[4,5], Federica Moalli[6], Francesca Mercalli[7], Maurilio Ponzoni[8], Rosanna Mezzapelle[1], Marina Ferrarini[3], Elisabetta Ferrero[3], Roberta Visone[2], Marco Rasponi[2], Marco E. Bianchi[1,8], Samuel Zambrano[1,8,*] and Alessandra Agresti[1,6*]

SUPPLEMENTARY FIGURES

Supplementary Figure 1: Detection of endogenous p65 in the BMs of MM patients.

(a) *IHC staining for p65 in a skin sample included in the BM biopsy of Pt.1. DAB, brown staining and Haematoxylin, blue. Red arrows: cells with NF-κB in the cytoplasm, with different staining degrees. Black arrows: basal follicle-stem cells with Nuclear NF-κB, as expected. White arrows: cells not expressing NF-κB. Scale bars: 10 and 50 µm.*

(b) *and* ***(c)****: additional examples of p65 IHC for BM samples from Pt. 1 and 2 to further support the conclusions from* ***Figure 1 panel a****.*

(d) *enlargement of the area included in the white rectangle in* ***Figure 1b*** *to detail p65 localization in MM cells from Pt.1. Other examples from 7 more patients are reported in* ***Supplementary Figure 1 part 2****.*

(e) *Cumulative and total frequency distribution of NCI values from* ***Figure 1f.***

(f) *IHC staining of FFPE Spongostan sections with two independent antibodies against p65 (Cell signalling and Thermofisher, Cat N° in the Methods section).*

Colombo et al., 2024, Supplementary figure 1

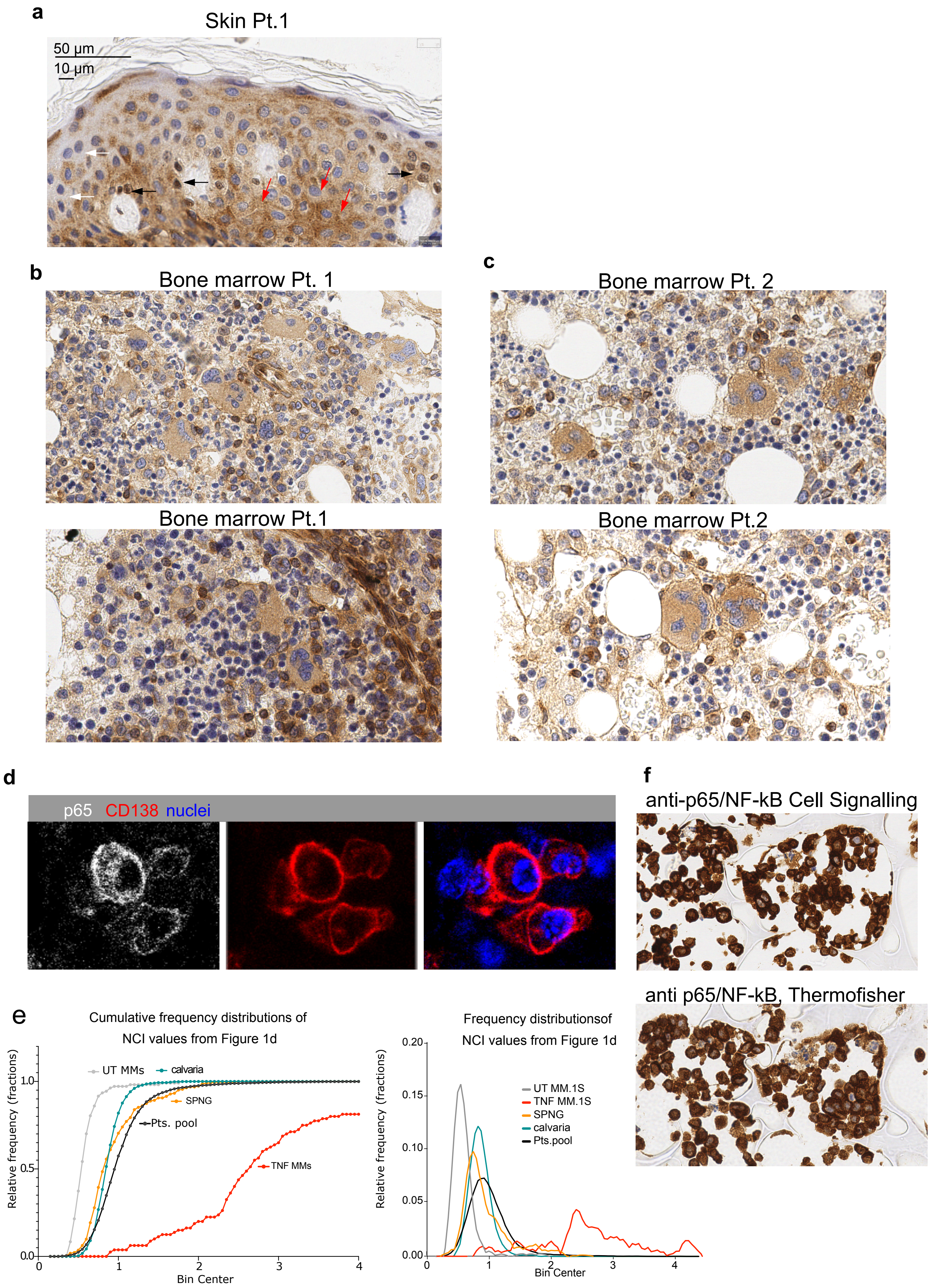

Supplementary Figure 1 part 2: Detection of endogenous p65 in the BMs of MM patients. *Details from IF staining of BM biopsies from 7 myeloma patients. Nuclei are in blue, p65/NF-κB in green, CD138 in white. Last panel contains the merge of the three channels. Scale bar 20 µm.*

Colombo et al., 2024, Supplementary Figure 1 part 2

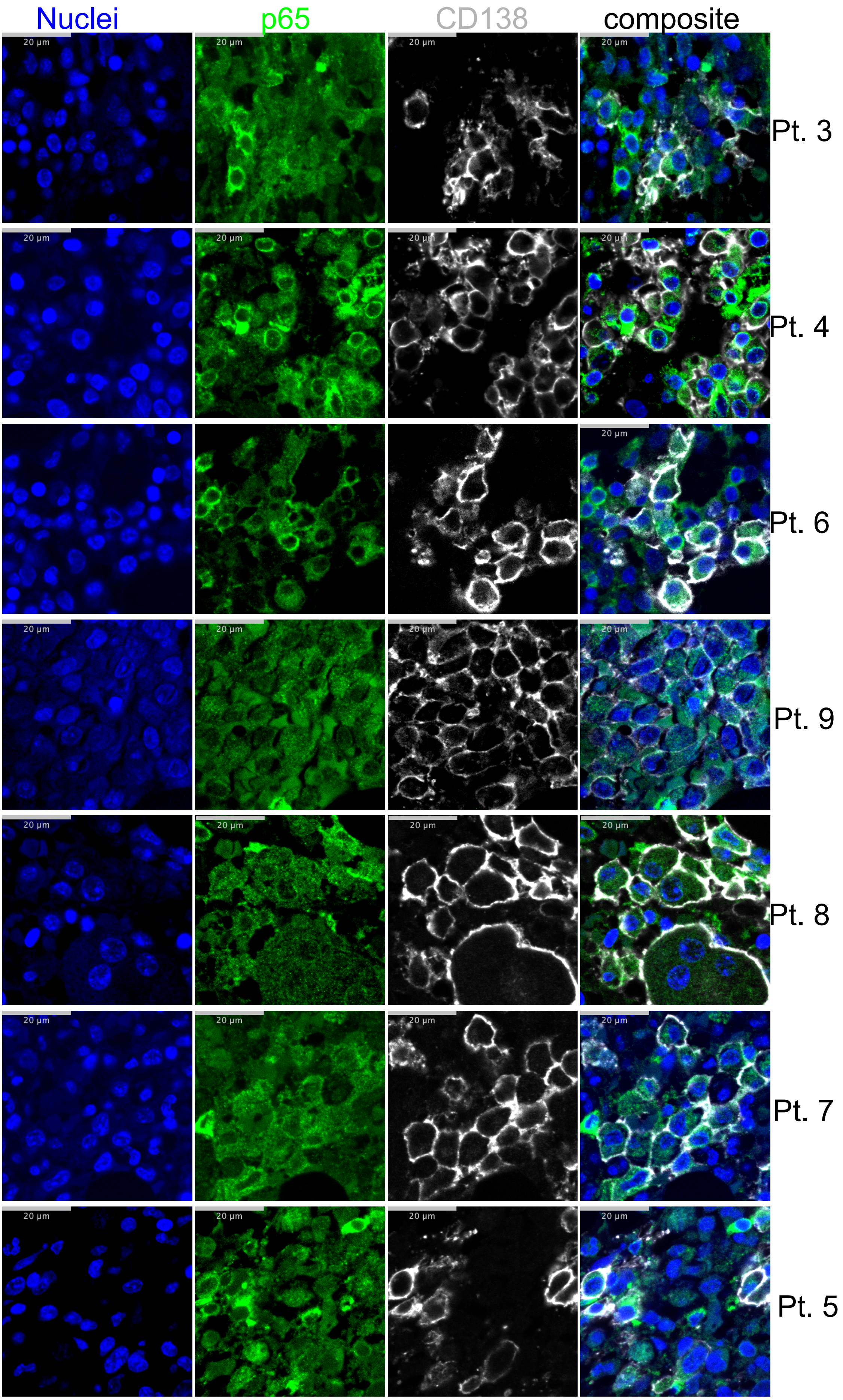

Supplementary Figure 2: Bioinformatics and mathematical modelling correlate MM cell genotype to NF-κB dynamics.

(a) *Unsupervised hierarchical clustering of 24 human MM cell lines based on their Copy Number Variations (CNVs) for genes involved in NF-κB signalling regulation. The colour scale bar on the right spans from "deep loss/homozygous deletion" (red) to "high level of gene amplification" (blue). The clustering subdivides the cell lines in three major groups with low, medium and high CNV burden (blue, green, red, respectively).*

(b) *Mathematical model for TNF-α driven signalling: gene transcription and negative feedbacks due to A20 and IkBa (TNFAIP3 and NFkBIA genes) repressors activity on the IKK signalling are reported* [16].

(c) *Mathematical modelling predictions for NF-κB dynamics as a function of time (x-axis) in a cell line bearing haploinsufficiency for the repressor IkBalpha like the MM.1S cells (red) and in normal non-mutated fibroblasts with physiological dynamics (blue).*

(d) *Mathematical model predictions when the expression of NF-κB regulatory genes is altered. Upper panel: loss-of-function mutations in the repressors A20 (red) or IkBalpha (black) genes lead to plateauing or sustained NF-κB activity, respectively. X-axis: time in hr.*
Lower panel: the NF-κB response is dramatically upregulated when the IKKα kinase activity is amplified by 2- (red), 10- (black) or 100- (blue) folds as compared to physiological dynamics (green). X-axis: time in hr.

Colombo et al., 2024, Supplementary Figure 2

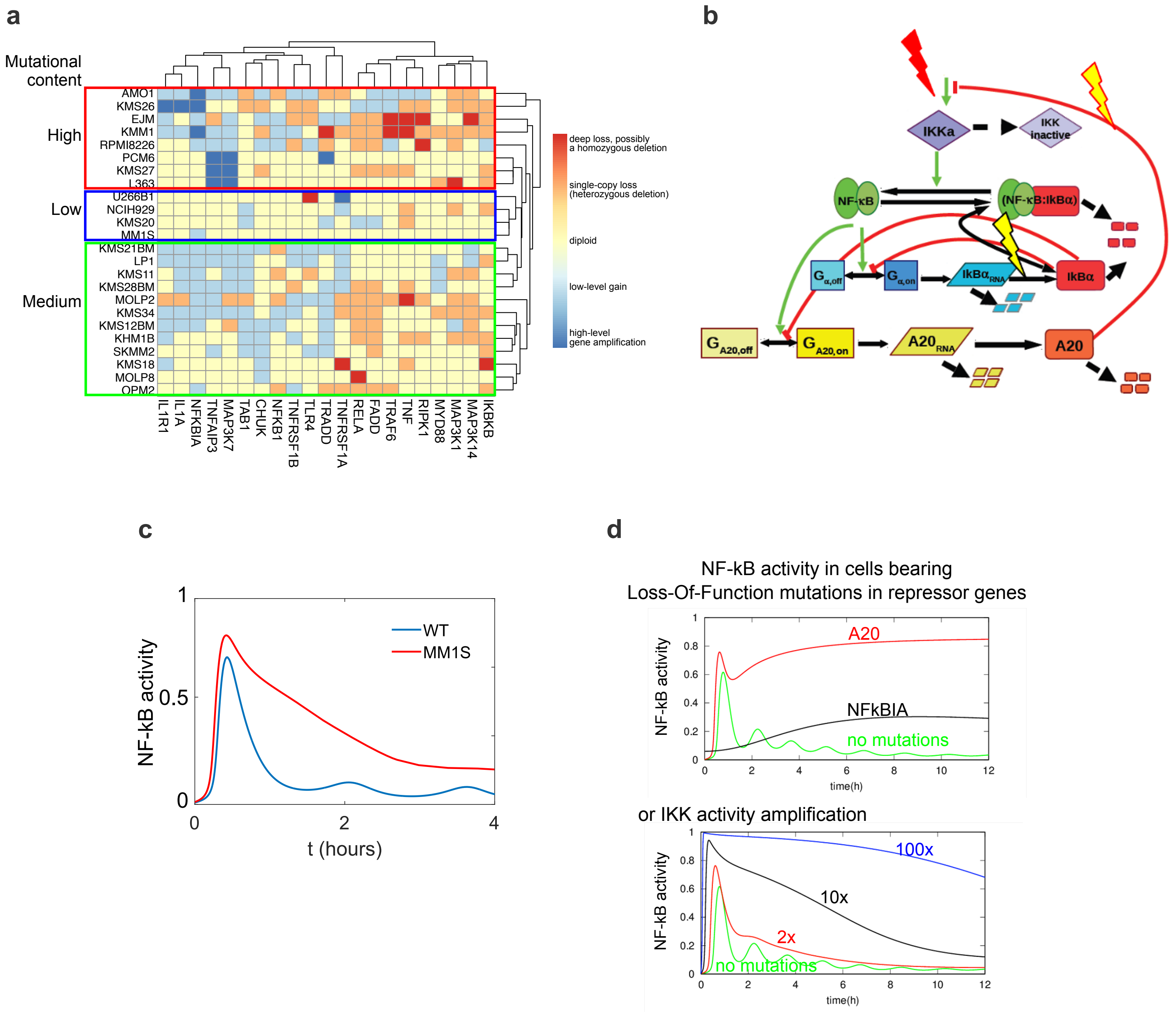

Supplementary Figure 3: IF for p65 shows quick TNF-α driven NF-κB activation in B cells followed by shut-down in 2 hours.

(a) *and* ***(b)*** *images show IF staining for p65 (white) in normal B cells* ***(a)*** *and the JY B-LCL cell line* ***(b)*** *treated for 0, 30, 60, 90, 120, 240 minutes with TNF-α. Nuclei are shown in blue in the composite panel. Scalebar: 20 µm.*

(c) *Note: the panel indexed with the "exposed as for B cells" title shows JY cells imaged with the microscope settings for Normal B cells to highlight the higher NF-κB content in JY cells. Scale bar: 20 µm in all the images. Of note, JY cells are much larger than normal B cells.*

(d) Biochemical quantification of p65 in HS-5, MM.1S and JY cells. *Cell lysates from the indicated cell types (HS-5, MM.1S and JY cells) were prepared at 0, 4 and 8 hours, either untreated (–) or TNF-α stimulated (+). Western blots were fluorescently immunostained for p65 and β-actin. Band intensities for p65 were quantified in high resolution, 16-bit images, normalised for β-actin band intensities and reported in the histogram. Note that MM.1S cells contain 2.5-fold more p65 than HS-5 cells. y-axis: actin-normalised p65 content; x-axis: cell types. Error bar: SD of two technical replicates from a representative experiment (unpaired t-test). Histograms on the bottom show the p65 fold change (y-axis) in the same cell types upon TNF-α stimulation. The amount of p65 protein in the three UT cell lines has been set to 1 to highlight no significant changes in p65 content upon TNF-α stimulation.*

Colombo et al., 2024, Supplementary figure 3

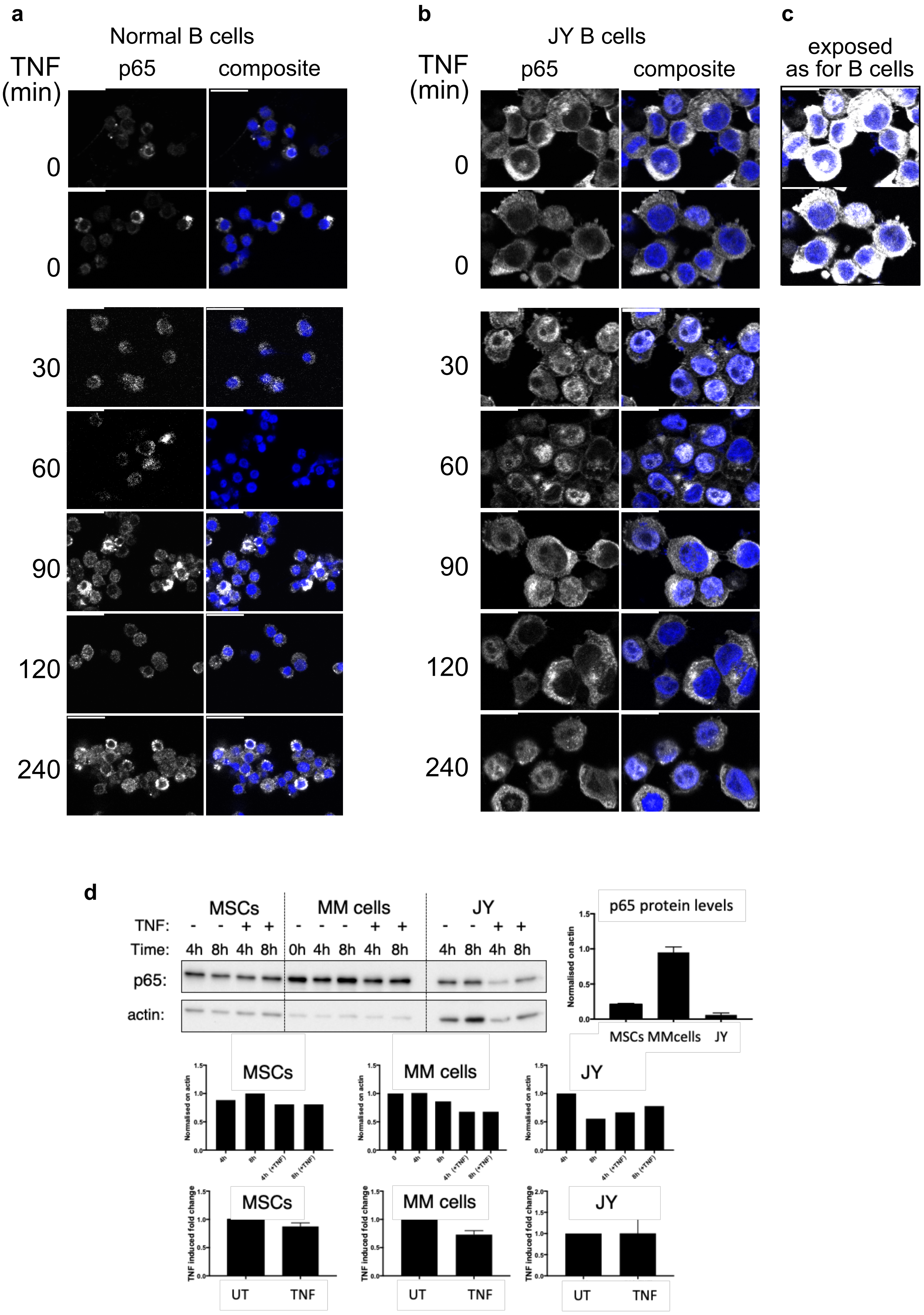

Supplementary Figure 4: MM cell lines show deregulated TNF-α driven NF-κB activation.

(a) *Confocal images of IF p65 staining (white, nuclei in blue) in cells treated with TNF-α for 0, 60, 120, 180 and 240 minutes. Scale bar: 20 μm.*

Cell lines: MM.1S, U266, OPM2 and RPMI8226. Note that OPM2 and U266 cells died after 60 and 120 min, respectively, of TNF-α stimulation.

(b) *The plot shows p65 NCI quantification in UT or TNF-α treated cells for 60min as illustrated in the images above. Box-and-whiskers represent 10-90% of the population, line is the median. + is the mean value. Statistical analysis by unpaired t-test, with Mann-Whitney test. Ns: not significant; * = 0.012; ** = 0.034; **** = <0.0001. "dead" indicates that U266 did not survive to the canonical 10 ng/ml TNF-α stimulation.*

Colombo et al., 2024 Supplementary Figure 4

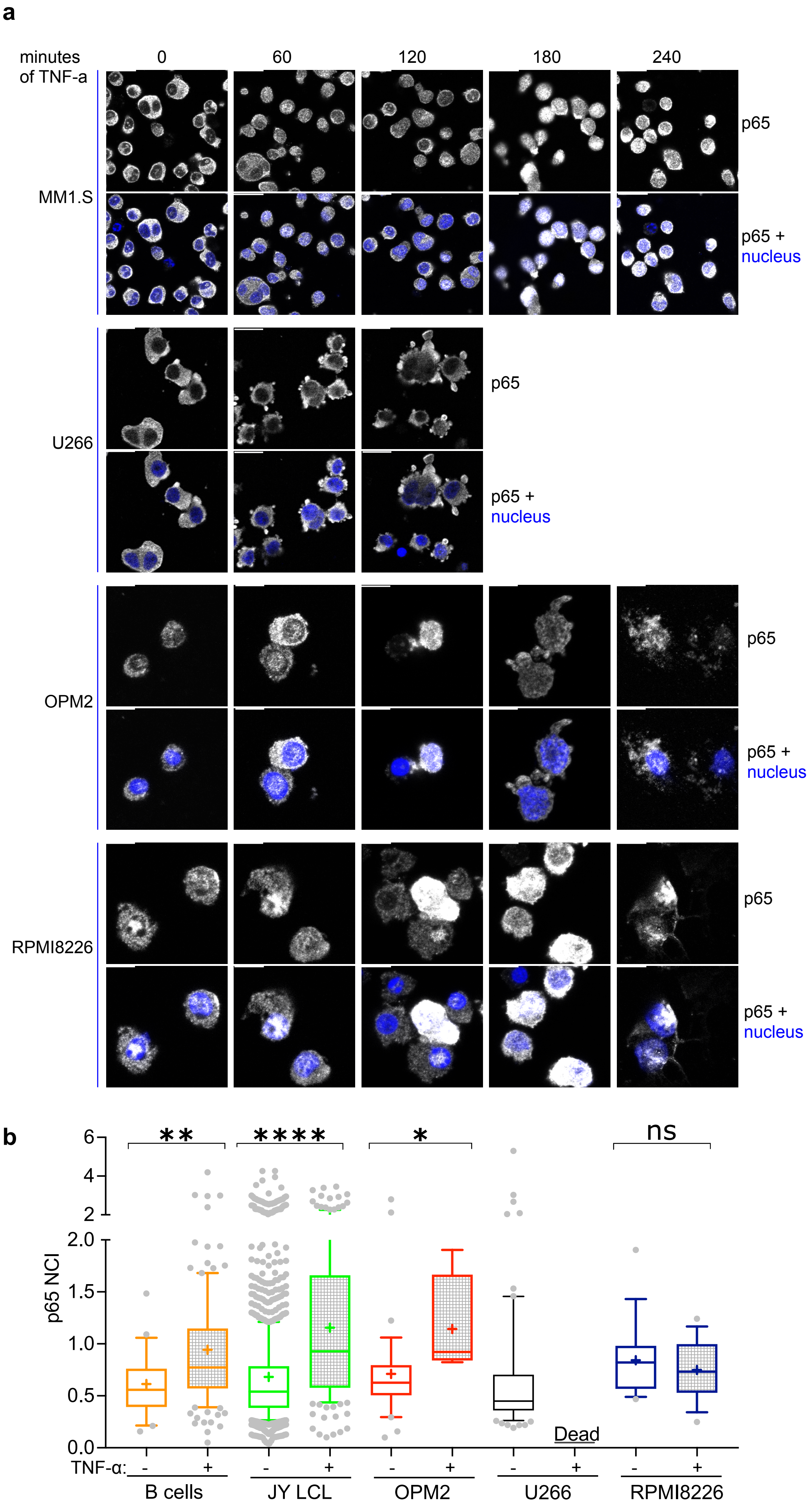

Supplementary Figure 5: TNF-α driven NF-κB dynamics in a collection of cell types.

(a) *BJ fibroblasts have been engineered to express YFP-p65 using the protocol defined for MM.1S cells reported in* ***Figure 2 panel a****. After sorting, p65-YFP BJ cells were stimulated or not with TNF-α and live imaged, as described in Methods section. In parallel, wildtype BJ cells were TNF-α treated, PFA fixed and IF stained for p65 as indicated.*

(b) *Endogenous p65 (IF, blue) and YFP tagged p65 (live imaging, orange) show similar probability of nuclear or cytoplasmic localization upon 60 minutes TNF-α treatment.*

(c) *Colorplots show p65 dynamics in untreated (left) or TNF-α treated (right) BJ fibroblasts.*

(d) *Quantification of the Area Under the Curve (AUC) in TNF-α treated BJ fibroblasts. Compare with AUC distribution in MM.1S cells in* ***Supplementary Figure 6f****.*

(e) *Colorplots of p65 NCI values obtained by TNF-α stimulation of a collection of human and murine YFP-p65 knock-in cell lines (HeLa, hu cervical carcinoma; Huh7, hu hepatocarcinoma; Mouse Embryo Fibroblasts; hu MM.1S myeloma cells). The red arrow and line indicate the timepoint for TNF-α stimulation.*

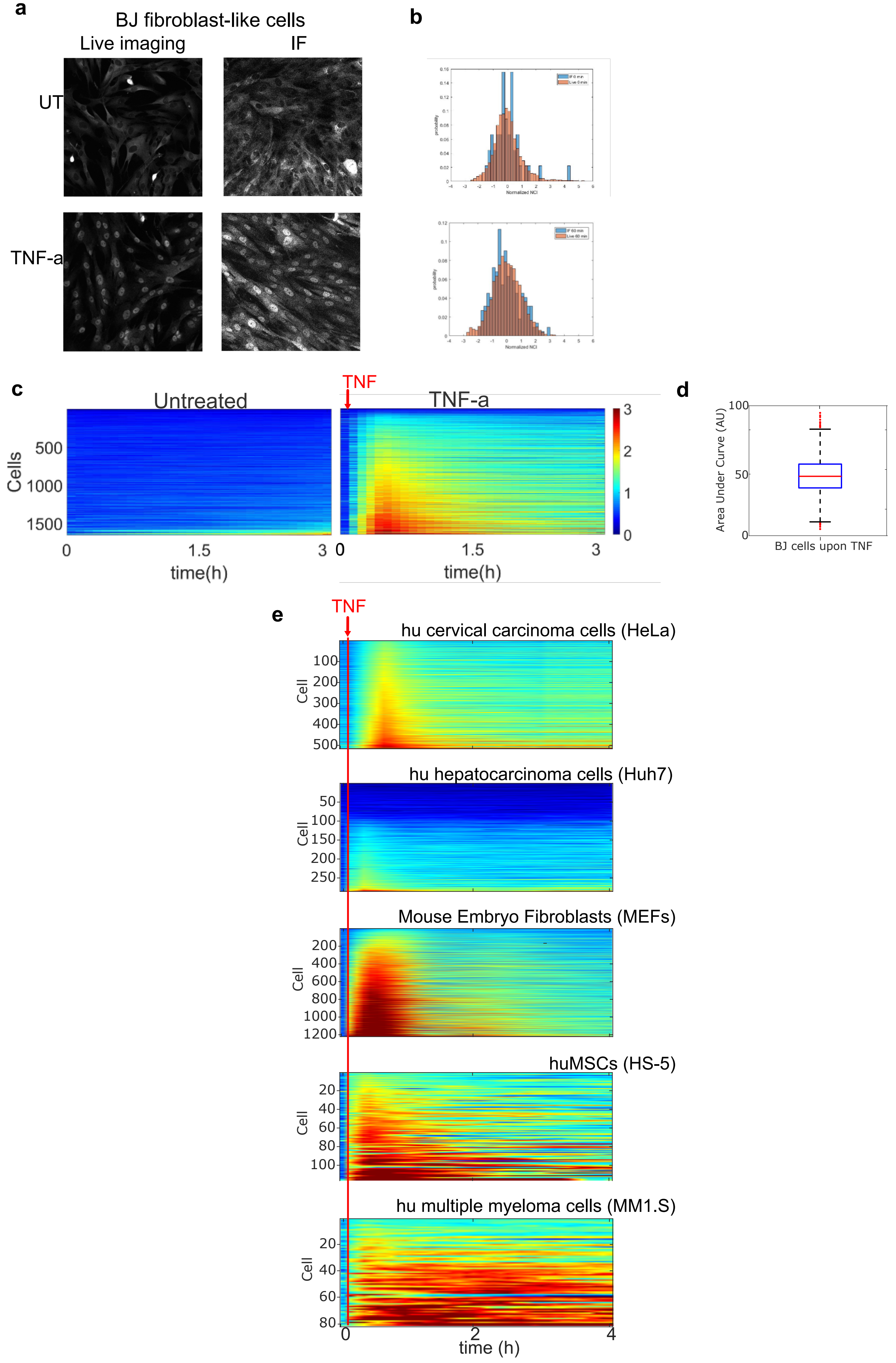
a
BJ fibroblast-like cells
Live imaging
IF
UT
TNF-a
b
Normalized NCI
c
Untreated
TNF
TNF-a
Cells
500
1000
1500
0
1.5
3
time(h)
d
Area Under Curve (AU)
100
50
0
BJ cells upon TNF
e
TNF
hu cervical carcinoma cells (HeLa)
Cell
hu hepatocarcinoma cells (Huh7)
Mouse Embryo Fibroblasts (MEFs)
huMSCs (HS-5)
hu multiple myeloma cells (MM1.S)
0
2
4
time (h)

Supplementary Figure 6: Characterization of MM.1S and HS-5 cells, both knock-in for YFP in the p65 locus

(a) Knock-in validation*: Left panel: Immunoblotting detects endogenous and/or YFP-tagged p65 protein in lysates from HS-5 and MM.1S cells either wildtype or p65-YFP knock-in as indicated. p65 knock-out MEFs do not show any p65 band. MW: molecular weight marker. Arrows indicate p65 (65 kDa), p65-YFP (90 kDa) and β-Actin (42 kDa) bands. Right panels: FACS analyses of p65-YFP knock-in MM.1S cells and HS-5 after sorting.*

(b) Static culture set-up*: Chambered coverslips used in live imaging.*

(c) Results of cell segmentation procedure *in unstimulated MM.1S cells and HS-5 used for p65 NCI quantification; such protocol has been applied to images from both live imaging and p65 IF.*

(d) YFP-tagged p65 and endogenous p65 protein respond similarly to stimuli. *Comparison of p65 NCI frequency distributions obtained by immunostaining (IF, blue histograms) and by live cell imaging (Live, orange histograms) at different time points after TNF-α stimulation (0, 60, 180 min) in wildtype and p65-YFP MM.1S cells, respectively. y-axis: frequency of NCI values; x-axis: NCI values.*

(e) Time integrated p65 activity (AUC) is a robust indicator for NF-κB activation in MM.1S and HS-5 cells*. Left: Intensity parameters are reported on an NCI profile from an MM.1S cell stimulated for 8 hours with TNF-α. Red line: NCI profile. Grey area corresponds to the "time-integrated activation" (Area Under the Curve, AUC); arbitrary NCI threshold= 1.2 (dashed line). The dotted black line indicates the 0.5fold NCImax to calculate the* T_{dec} *(see below).*

(f) Violin plots compare AUC distributions *in a representative experiment out of 3 performed. Significance between AUC distributions was assessed by ANOVA/Kruskal-Wallis test for multiple comparisons (P<0.0001, **** and 0.001, ***). Results are comparable to NCI distribution shown in* ***Figure 2.***

Colombo et al., 2024, Supplementary Figure 6

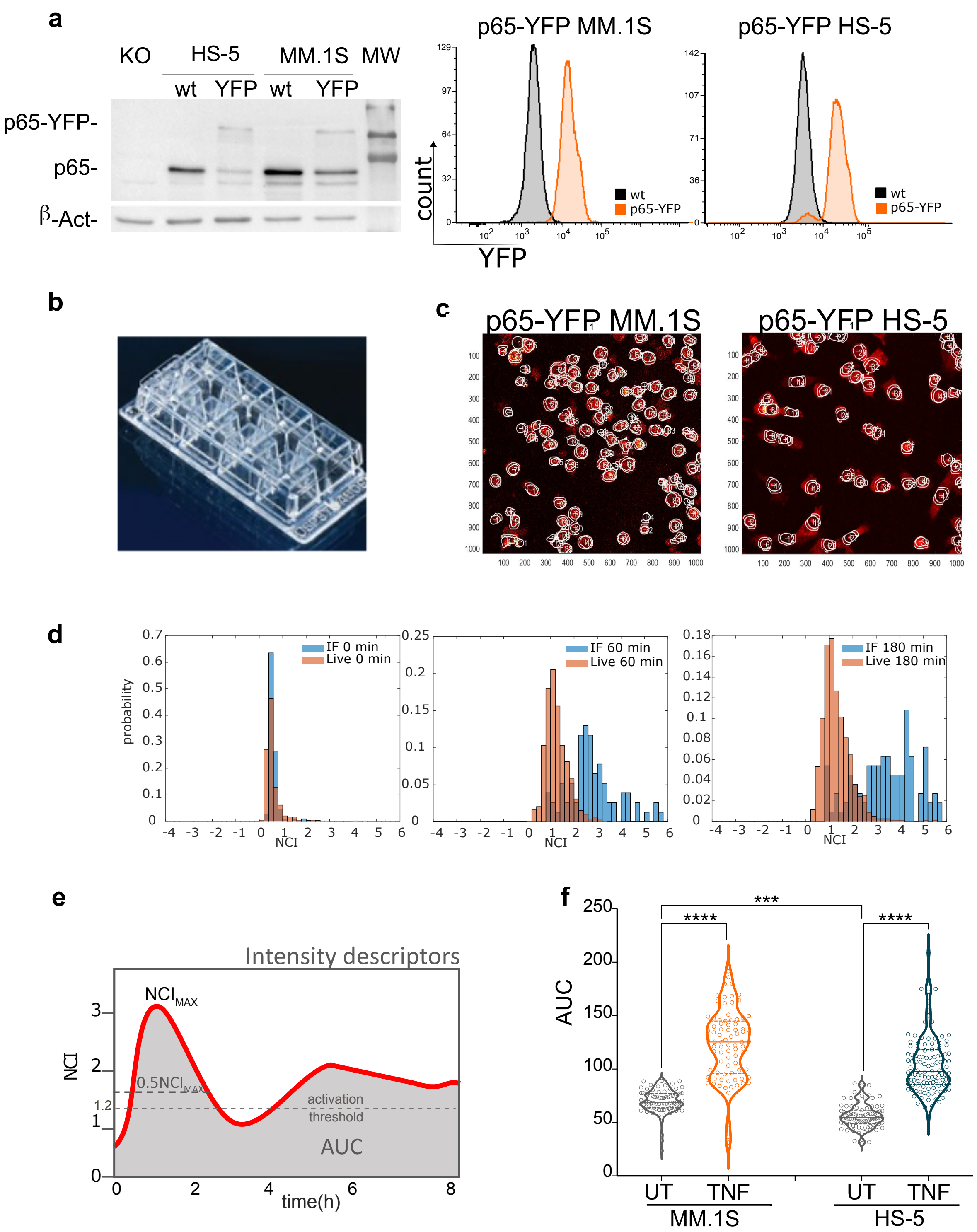

Supplementary Figure 7: Heterogeneous responses to TNF-α in HS-5 and MM.1S cells in static cultures

(a) *Box plots compare NCI distributions in three independent experiments with static cultures of p65-YFP MM.1S cells and p65-YFP HS-5 as indicated.*

(b) *Collection of NCI tracks for the cells analysed in* **Figure 2***. Each thin line corresponds to a single cell. MM.1S in red, HS-5 in blue, either UT or TNF-α treated.*

(c) *Unsupervised K-mean clustering of the tracks in* **panel b.** *Percentages indicate the cell fraction in Cluster 1, 2 and 3 over the total population.*

(d) *Statistical analyses of cluster differences.*

Colombo et al., 2024, Supplementary Figure 7

a: Reproducibility in static cultures

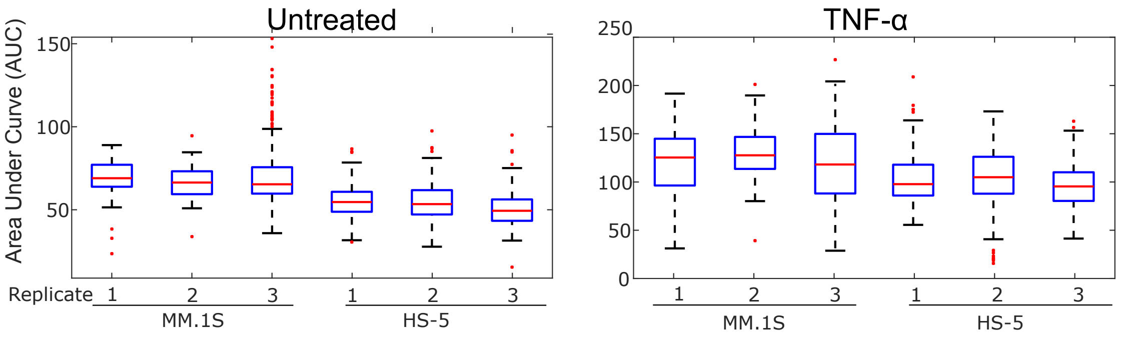

b: single cell tracks

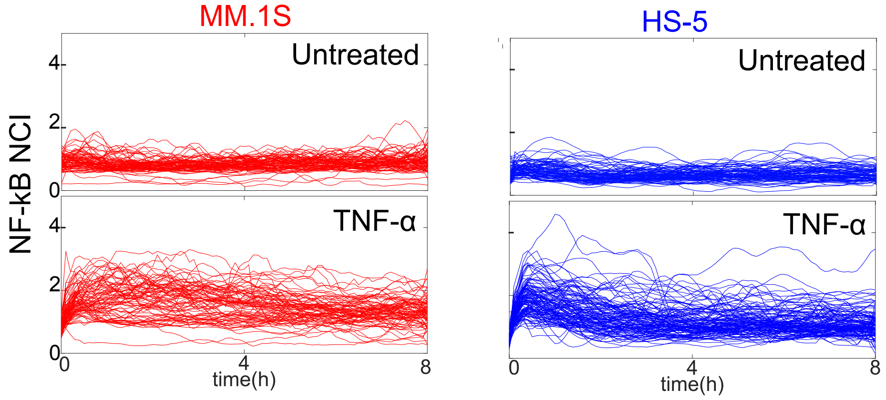

c: Unsupervised K-mean clustering

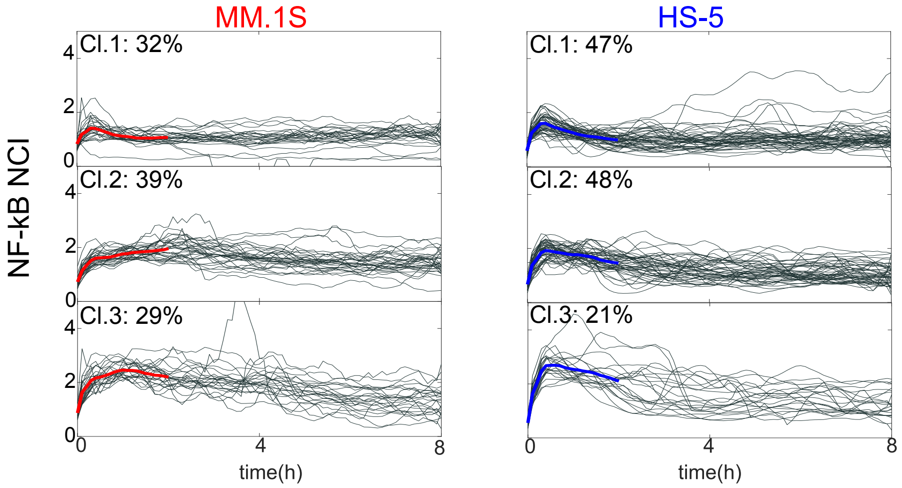

d: Statistical analyses of cluster differences

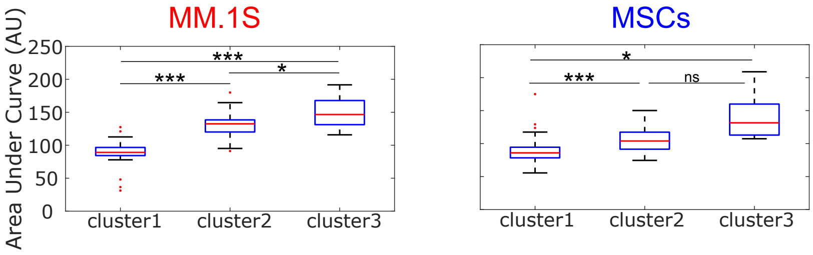

Supplementary Figure 8: Microfluidic set up to remove autocrine/paracrine signals possibly affecting p65 dynamics when HS-5 and MM.1S cells are treated or not with TNF-α.

(a) *Microfluidic set-up, modified from CellASIC^{TM} ONIX Microfluidic Platform (https://www.merckmillipore.com/IT/it/product/CellASIC-ONIX-Microfluidic-Platform,MM_NF-C117908 and [16])*

(b) *and* ***(c)*** *Images of cells plated in round microfluidic chambers and treated as indicated. Scale bar 50 µm.*

(d) *Box-and-whisker plots of time parameter distributions (T_{max}, T_{act} and T_{dec}) for p65 activation in microfluidic-plated HS-5 and MM.1S cells analysed in* ***Figure 2.***

(e) *comparison p65/NCI outputs from TNF-α and IL-1β stimulated MM.1S and HS5 in flow-cultures.*

Colombo et al., 2024 Supplementary Figure 8

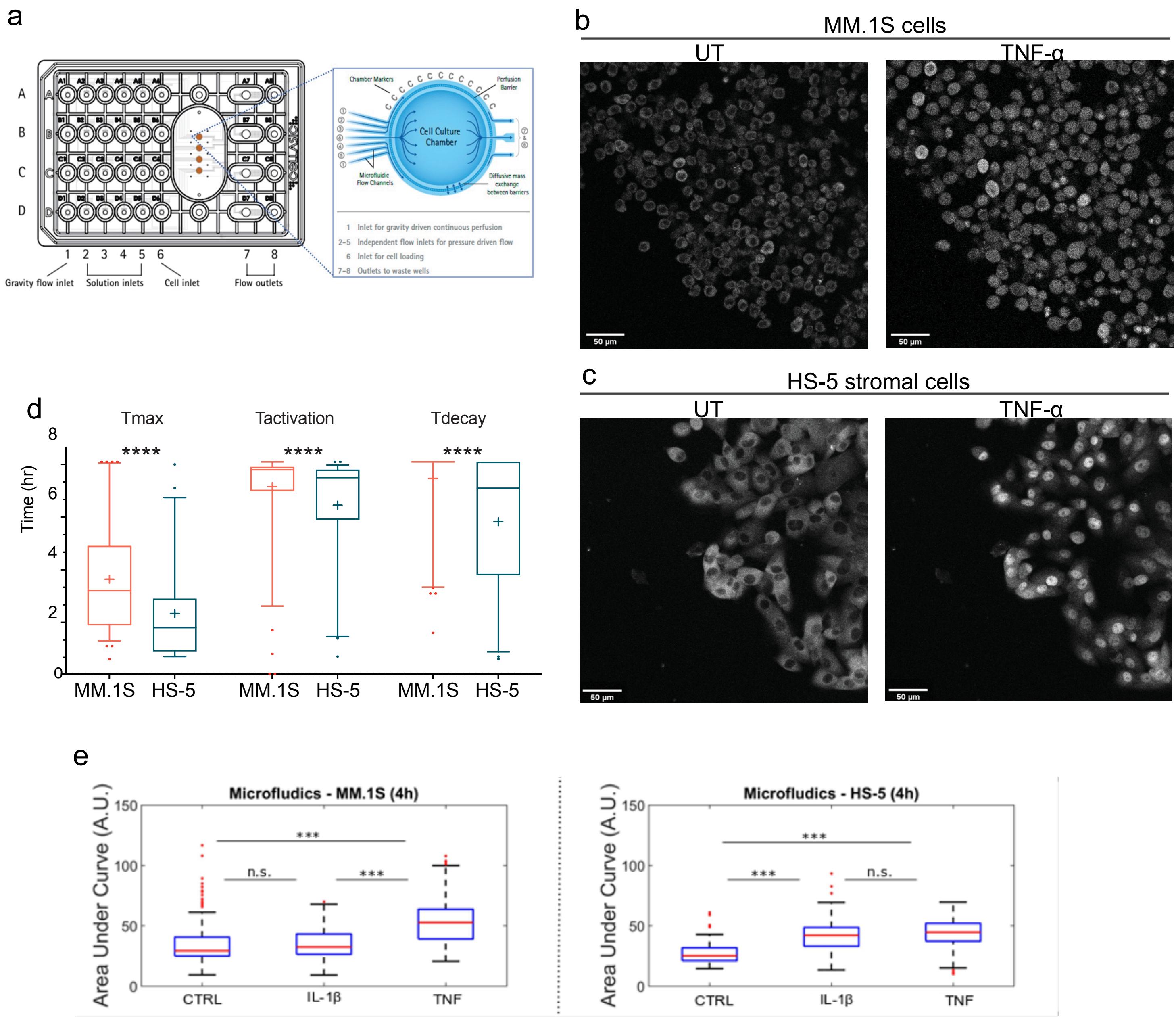

Supplementary Figure 9: Cell density and medium flow do not affect NF-κB basal levels in MM.1S and HS-5

(a) and (b)*: MM.1S and HS-5 cells are plated at low density and exposed to medium flow for 1 hour. The four colorplots are from 2 independent experiments.*

(c) and (d)*: as above with cells at higher 2D plating density.*

Colombo et al., 2024, Supplementary Figure 9

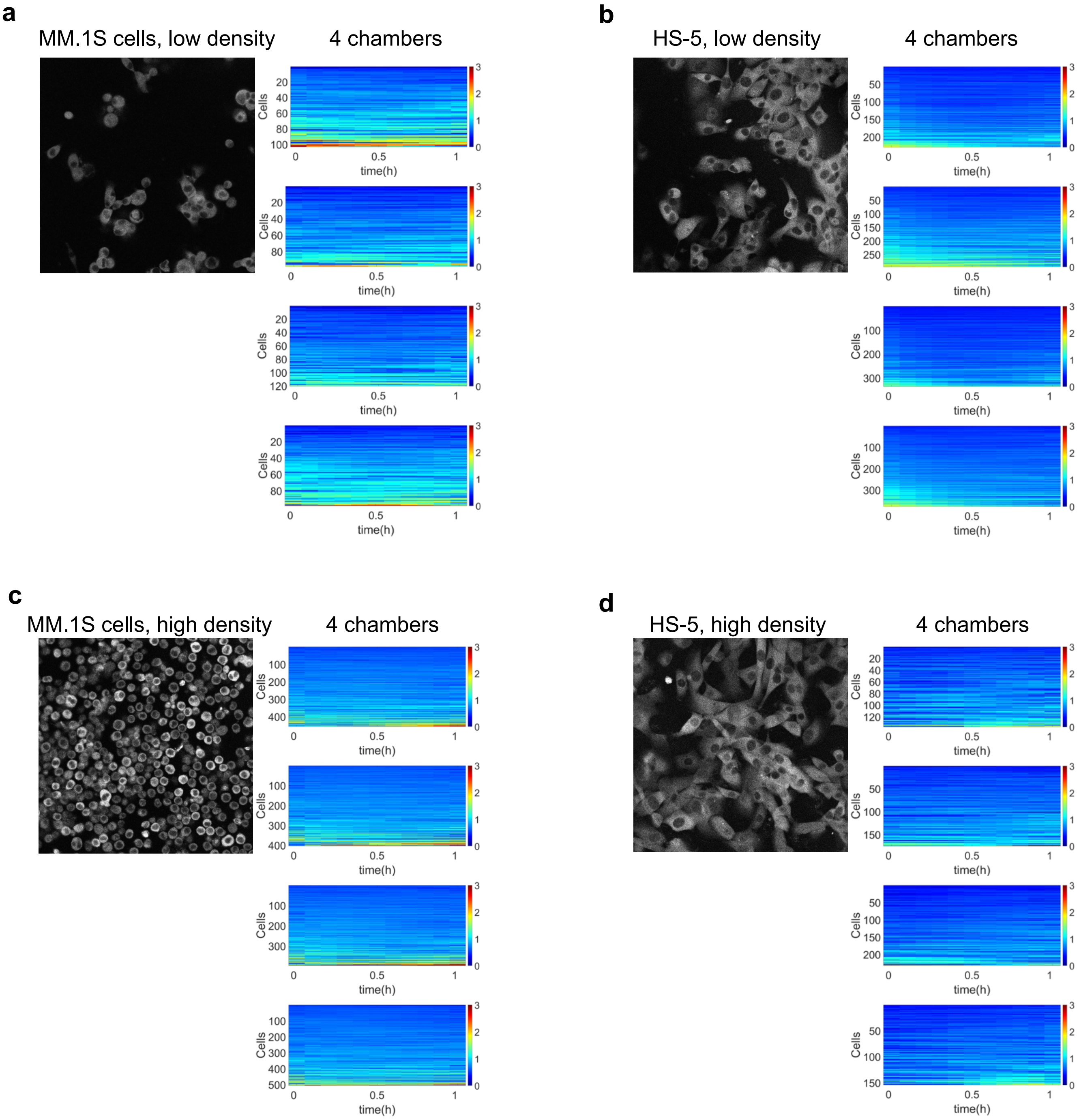

Supplementary Figure 10: Heterogeneous responses to TNF-α in HS-5 and MM.1S cells in microfluidic chambers

(a) *Collection of NCI tracks obtained from cells analysed in **Figure 3**. Each thin line corresponds to a single cell.*

(b) *Unsupervised K-mean clustering of the tracks in **panel a**. Percentages indicate the cell fraction over the total population in Cluster 1, 2 and 3.*

(c) *Statistical analyses of cluster differences by ANOVA + Tukey's multiple comparisons test (****, P<0.0001).*

a: Single cell tracks in cultures upon flow

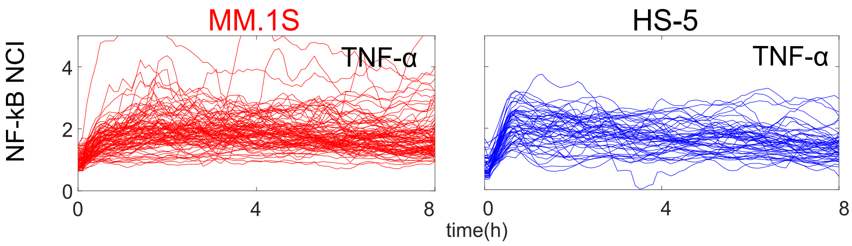

b: Unsupervised K-mean clustering

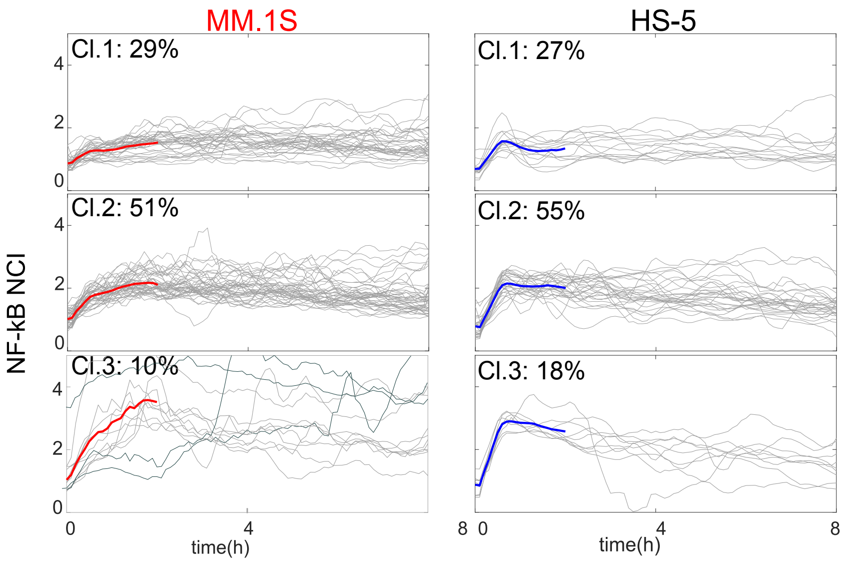

c: Statistical analyses of cluster differences

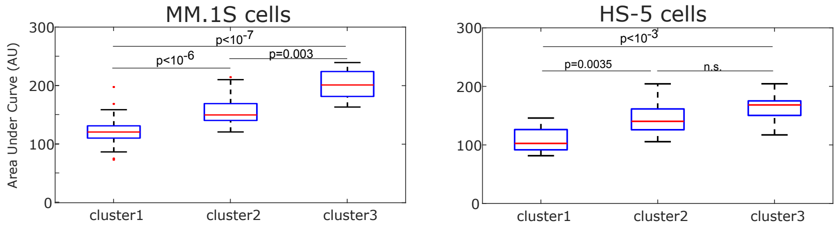

Supplementary Figure 11: Additional data related to the quantifications of autocrine/paracrine signals.

(a) Cytokine and Chemokine quantification in MM.1S cell supernatants using the Proteome Profiler Human XL Cytokine Array Kit, R&D Systems*. Filters incubated with supernatants as indicated were stained with a Cy-5 labelled Fluorescent secondary antibody. Fluorescence was acquired as high-res 16bit images. Rectangles indicate the positions of the three internal positive controls used for normalization (red), and the TNF-α spots (green), in all the filters. Two independent quantifications, with two technical replicates each, were performed. One representative filter for each condition is shown.*

(b) CK levels in MM.1S cells are minimally affected by TNF-α stimulation. *y-axis: fold change of CK levels in TNF-α treated vs UT cells. Error bars: SD of two technical replicates and 2 independent experiments.*

(c) Detection and quantification of CKs secreted by HS-5 upon stimulation with TNF-α *as described for panel* ***a*** *above.*

(d) CK levels in HS-5 are affected by TNF-α stimulation: *plot as described in panel* ***b.***

(e) Detection and quantification of CKs secreted by HS-5 upon stimulation with IL-1β *as described for panel* ***a*** *above. The purple rectangles highlight the IL-1β spots.*

(f) CK levels in HS-5 are affected by IL-1β stimulation: *plot as described in panel* ***b.***

(g) Secretory profile in HS-5 upon IL-1β stimulation. *Histogram represents CKs quantification in supernatants from untreated (grey bars) or IL-1β stimulated HS-5 (magenta) for two hours perform as described for* ***Figure 4 a****. Bars represent normalised fluorescent signals averaged from two independent experiments in technical duplicates. Error bars: SD. y-axis: normalised fluorescence intensity in AU. x-axis: detected CKs.*

(h) Split UMAP representation of the transcriptional analysis for MM.1S and HS-5 populations alone or in coculture. *Clusters reported in* ***Figure 4 panel e*** *are here represented separately based on their cell identities to better identify subpopulations: HS-5 alone (cluster 0, dark green), upper left; HS-5 in coculture with MM.1 cells (cluster 2, teal), upper right. MM.1S cells alone clusters 1 and 3, dark red and orange), lower left; MM.1S cells in coculture with HS-5 (cluster 4, yellow), lower right. Colour codes as in* ***Figure 4 panel e.***

(k) Pathways analyses in MM.1S cells and HS-5 in mono- or co-culture. *Table reports the Bioplanet 2019 pathways that are expressed at basal level and after 2-hour cocultures. For each pathway, the P-value, Adj.P-value, Odds ratio and Combined score calculated by EnrichR are reported.*

(i) *In the left panel, UMAPs show the transcription levels of IL1B, IL1A, IL1R1 and IL1RN genes in the four populations shown in panel* ***(h)****. The purple arrow "only positive cells" points to the same panels on the left but devoid of all the cells that are negative for each specific gene (grey dots covering the purple ones).*

Colombo et al., 2024, Supplementary Figure 11

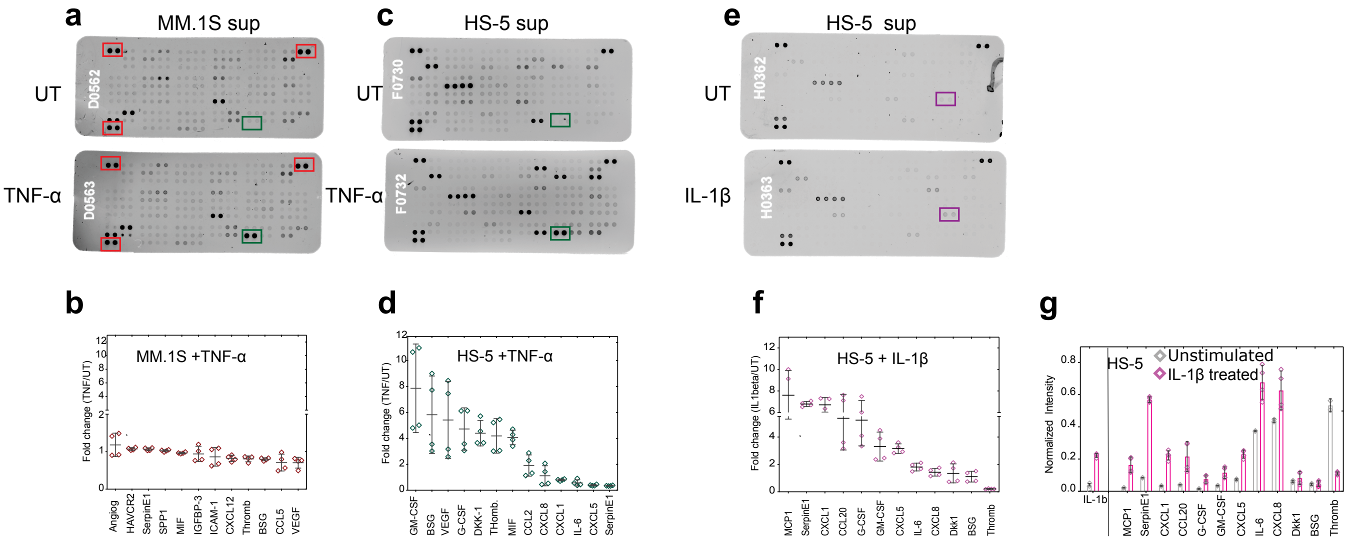

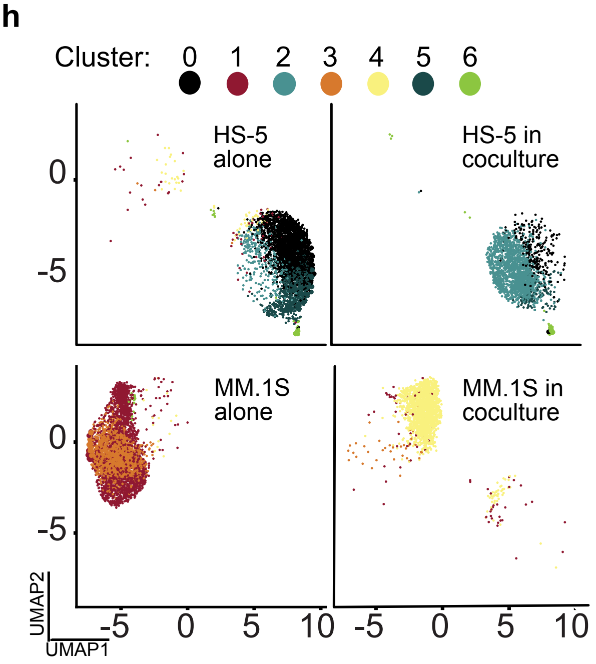

k

MM1.S cells alone

Pathway	P-value	Adj-P value	Odds ratio
Interferon alpha/beta signaling	5.59E-17	9.05E-15	118.91
Immune system signaling by IFNs, ILs, and GH	2.47E-13	1.33E-11	30.40
Immune system	5.26E-08	1.50E-06	8.94
Type II interferon signaling	5.57E-08	1.50E-06	61.46
Activation of DNA fragmentation factor	5.93E-05	4.21E-03	226.94

MM.1S cells in coculture with HS-5

Pathway	P-value	Adj-P value	Odds ratio
BDNF signaling pathway	2.27E-13	8.46E-12	7.86
T cell receptor regulation of apoptosis	6.31E-13	2.20E-11	4.92
Protein export	2.44E-08	6.49E-07	30.28
Antigen processing and presentation	1.63E-07	3.97E-06	10.45
Immunoregulatory interactions between a lymphoid and a non-lymphoid cell	3.99E-05	7.43E-04	6.03

HS-5

Pathway	P-value	Adj-P value	Odds ratio
Electron transport chain	8.85E-41	5.25E-38	42.55
BDNF signaling pathway	3.19E-12	1.82E-09	8.18
NF-kappaB activation	2.93E-05	9.81E-04	87.08
ECM-receptor interaction	3.14E-05	6.89E-04	61.43
Integrin cell surface interactions	3.25E-05	6.89E-04	60.68
Inflammatory response pathway	2.26E-04	3.89E-03	109.65

MSCs in coculture with MM.1S cells

Pathway	P-value	Adj-P value	Odds ratio
Cell cycle	2.93E-27	2.96E-24	5.88
DNA replication	3.86E-19	1.95E-16	7.30
T cell receptor regulation of apoptosis	2.19E-16	4.41E-14	3.75
Activation of NF-kappaB in B cells	3.83E-11	3.22E-09	10.18
Apoptosis	3.31E-10	1.96E-08	4.38
TGF-beta regulation of extracellular matrix	4.36E-10	2.44E-08	2.98

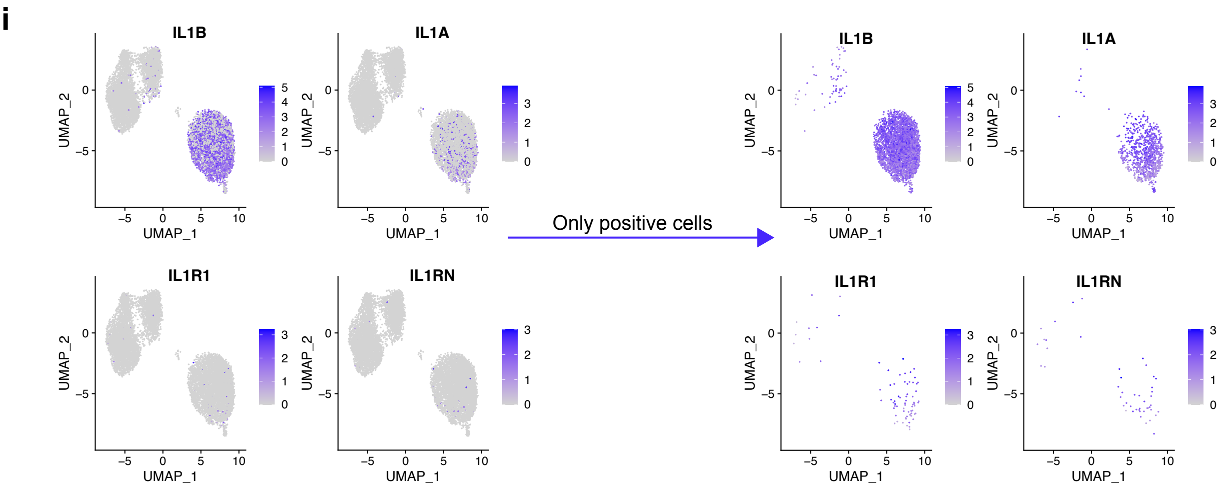

Supplementary Figure 12: Description of custom microbioreactors

(a) Layout of microbioreactor construction, culture, pressure-actuated valves and combined layers. *Left panel, the culture layer composed by two symmetric culture microchambers and side channels for medium delivery. The red rectangle contains a blow up of the boundaries between culture and medium microchamber separated by pillars: blue background, inner culture chamber loaded with cell-laden fibrin; pink background, side channels used for medium delivery. Central panel: pressure actuated compartment composed of 4 round closed valves, in red. Right panel, the combined layout from superimposed layers.*

(b) Evaluation of the minimal negative pressure for valves actuation to connect culture chambers. *Valves were imaged with a microscope and representative pictures from one experiment are shown. The intensity of the negative pressure applied to open the valves is reported in mmHg. The side panel shows the ROIs (Regions-Of-Interest) used for quantification. Scale bar: 500 µm.*

(c) The plot correlates the negative pressure *applied to the valves in mm Hg (x-axis) and the mean intensity of the ROI in the channel (error bars: SD from N=3 independent microbioreactors)*

(d) Effective chamber separation and rapid diffusion time. *The cartoon shows the upper side-channel loaded with a green fluorescent molecule (MW 10 kD) injected with closed valves.*

(e) *Panels show the absence of leakage between delivery channels and culture chambers, up to 8 hours: fluorescence is detected on one side of the valve (1 left) but not in the cell-laden fibrin compartment (2 left) and in the opposite cell chamber (3 left). Upon valve opening (right panels), the green fluorescence is detected almost immediately at position 1, after 4 min in the culture channel center (2) and, after 8 min, in the culture compartment.*

(f) NF-κB translocation induced by a panel of cytokines in MM.1S and HS-5 cells. *Upper panel: confocal images of MM.1S and HS-5 cells in static chambers, either untreated or treated for 45 minutes with the indicated CKs. Scale bar: 50 µm. Box plots below summarise NCI distributions for MM.1S cells and HS-5 cultured in static chambers. Statistical significance by Kruskal- Wallis test ($P<0.0001$) is summarised in the tables on the right.*

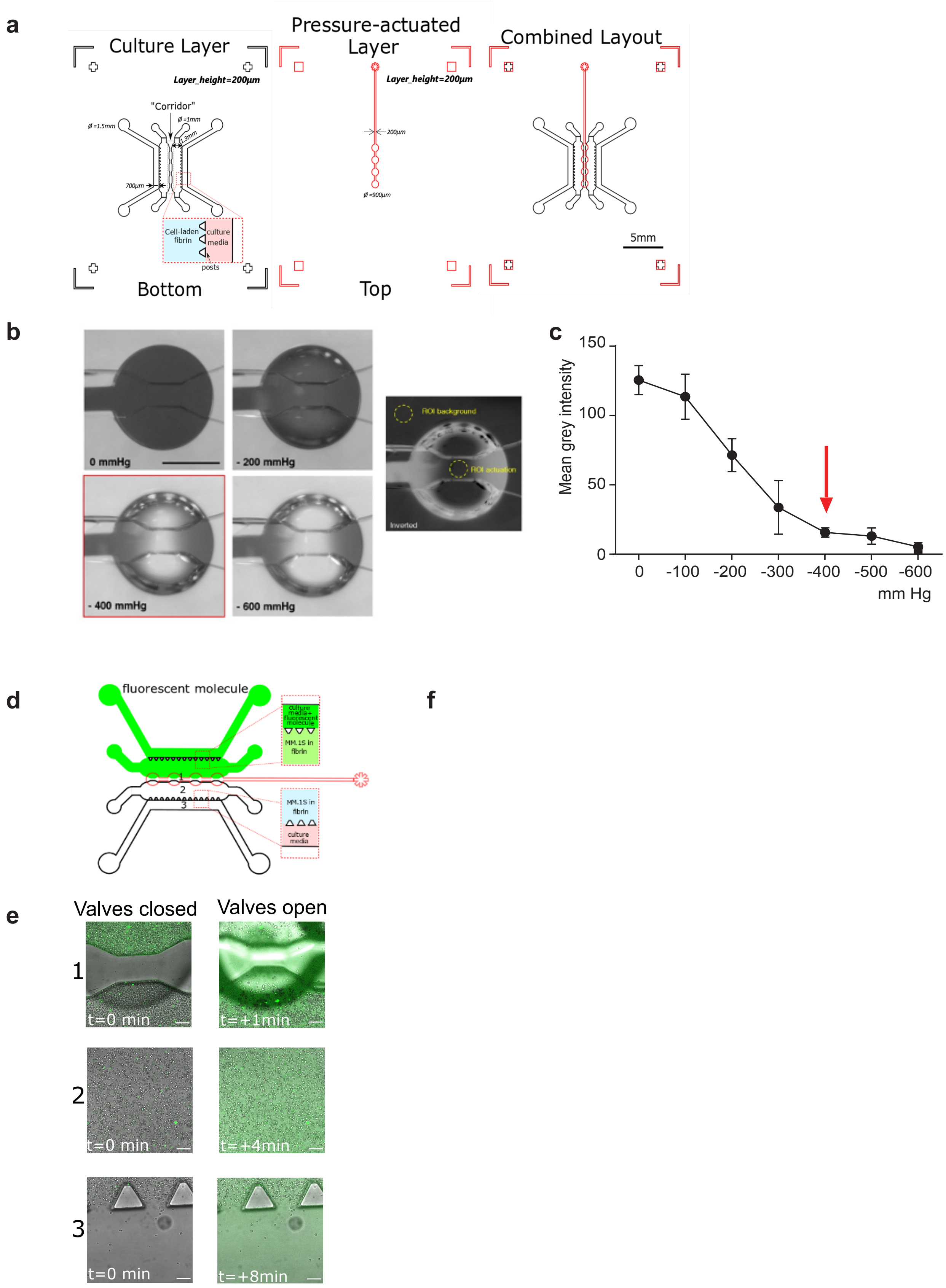
a
Culture Layer
Layer_height=200µm
"Corridor"
Cell-laden fibrin
culture media
posts
Bottom
Pressure-actuated Layer
Layer_height=200µm
200µm
Top
Combined Layout
5mm
b
0 mmHg
- 200 mmHg
- 400 mmHg
- 600 mmHg
ROI background
ROI actuation
Inverted
c
Mean grey intensity
mm Hg
d
fluorescent molecule
MM.1S in fibrin
culture media
f
e
Valves closed
Valves open
t=0 min
t=+1min
t=+4min
t=+8min

Supplementary Figure 13: Set up to evaluate MM.1S-HS-5 crosstalk in custom 3D microbioreactors.

(a) *Images of HS-5 plated in the upper chamber of the microbioreactor after 2 hr IL-1β stimulation (position 1 and 3 in panel b). Note the nuclear localization of p65-YFP. Scalebar: 50 µm.*

(b) *Diagram of the microbioreactor. The imaged fields, partially shown in* ***panel d****, are numbered from 1 to 10. Scalebar: 5mm.*

(c) *Confocal images of the cells plated in the microbioreactor before imaging. Note the empty nuclei in the enlarged circles.*

(d) *Confocal images of fields 4, 6, 8 and 10 showing MM.1S cells before (left column, valves closed) and after 2 hr exposure to IL-1β CM from HS-5 (valves open). The third panel to the right is an overexposed confocal image of position 4 to appreciate the design of pillars and channels. In timelapse imaging, cells are tracked by a dedicated software (Methods section) and can be followed along time. Each red arrow points to the same cell before and after stimulation. Nuclear NF-κB can be appreciated. Scale bar: 50µm*

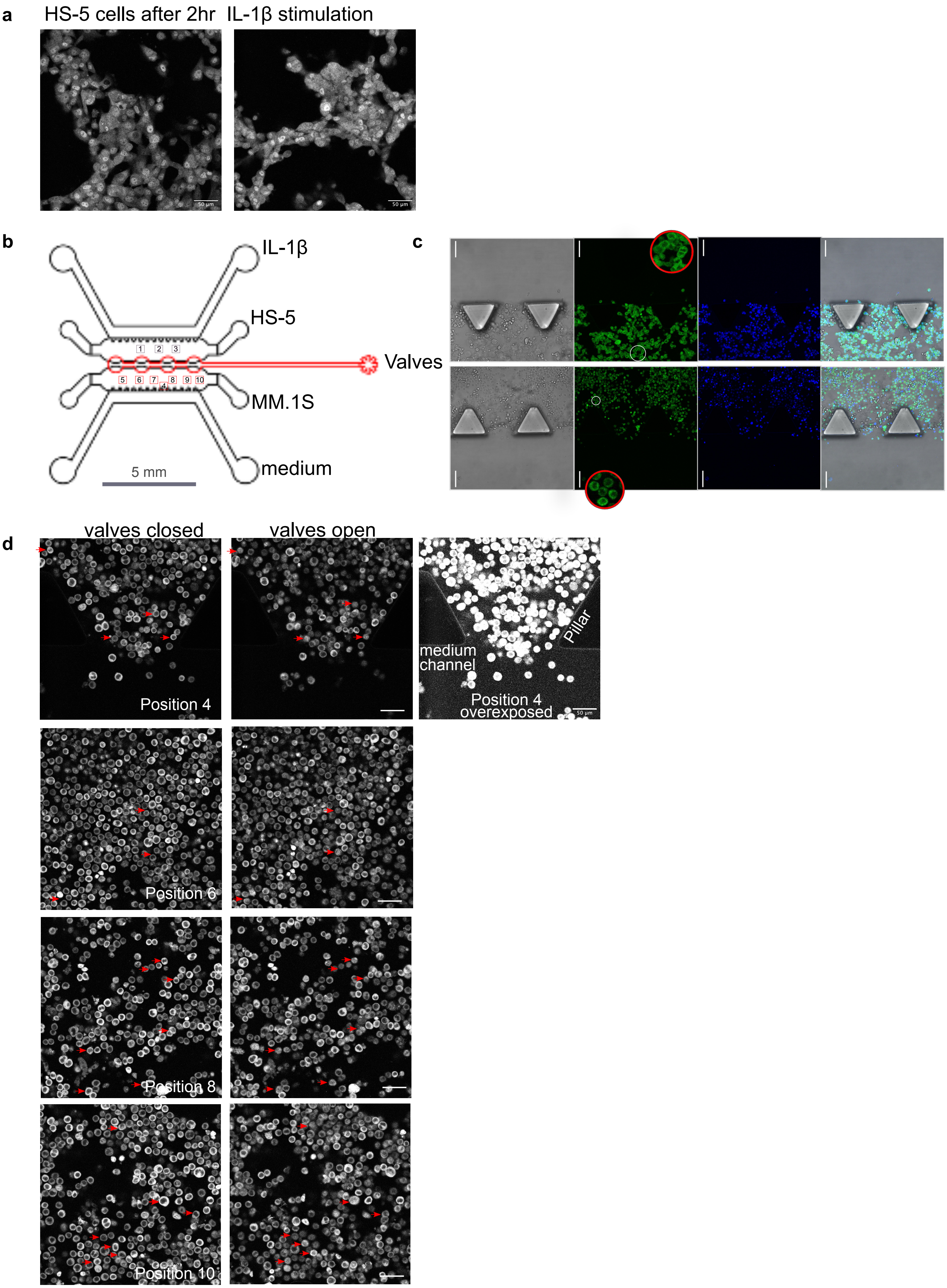
a
HS-5 cells after 2hr IL-1β stimulation
b
IL-1β
HS-5
1 2 3
5 6 7 4 8 9 10
Valves
MM.1S
medium
5 mm
c
d
valves closed
valves open
Position 4
medium channel
Pillar
Position 4 overexposed
Position 6
Position 8
Position 10

Supplementary Figure 14: IL-1β-driven activation of NF-κB in the BM

*Cumulative **(a)** and total frequency **(b)** distributions of NCI values from data represented in **Figure 6 panel e.***

***(c)** Violin plots compare NF-κB activation (NCI) in MM.1S cells engrafted in the calvaria of mice either untreated or treated for 3 hours with TNF-α or IL-1β, or 24 hr with Anakinra (IL-1β inhibitor). The distribution includes NCI values within the 25% and 75% limits NCI distribution of the total population.*

*This analysis complements those in **Figure 6, panel f** with the top 25% distributions. Statistical analyses by ANOVA with Dunn multiple comparison test: significance as indicated in the plot: ****p<0.0001, ns: not significant).*

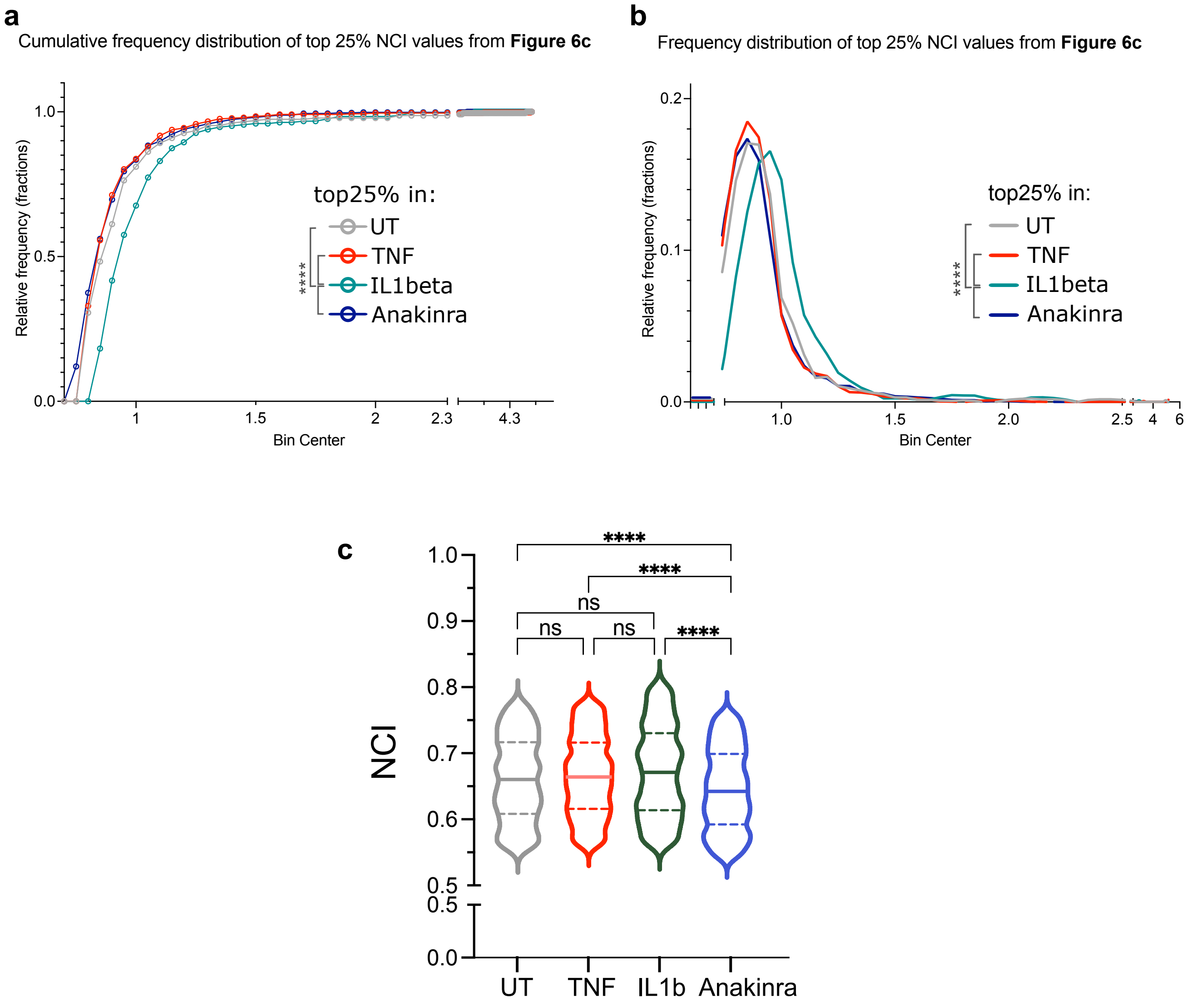
a
Cumulative frequency distribution of top 25% NCI values from Figure 6c
Relative frequency (fractions)
1.0
0.5
0.0
1
1.5
2
2.3
4.3
Bin Center
top25% in:
UT
TNF
IL1beta
Anakinra
****
b
Frequency distribution of top 25% NCI values from Figure 6c
Relative frequency (fractions)
0.2
0.1
0.0
1.0
1.5
2.0
2.5
4
6
Bin Center
top25% in:
UT
TNF
IL1beta
Anakinra
****
c
NCI
1.0
0.9
0.8
0.7
0.6
0.5
0.5
0.0
****
****
ns
ns
ns
****
UT
TNF
IL1b
Anakinra

Title:

In vitro models of the crosstalk between multiple myeloma and stromal cells recapitulate the mild NF-κB activation observed in vivo.

Supplementary information to each figure

FIGURE 1

NF-κB activation in BM-dwelling MM cells is mild and heterogeneous

Criterions for the identification of myeloma cells in the patients' bone marrow:

MM cells are round, with eccentric nuclei, and are larger than the erythroid precursors[1].

The nucleus is large, sometimes haematoxylin staining of chromatin highlights a "cart-wheel" chromatin configuration[2], right.

Such large cells are often bi- or multinucleated.

Cell membranes are positive for CD138 in Immunofluorescence staining.

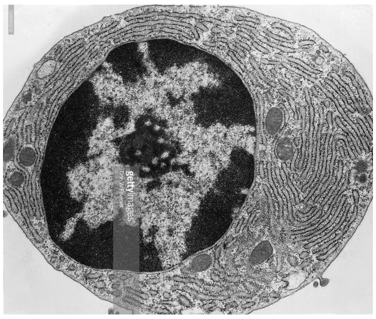

Snapshots below are from anti p65 IHC staining (brown) in of BM from Patient 1, 2 and 5 (see Figure 1 and Supplementary Figure 1). They show the typical nuclear cartwheel staining in myeloma cells upon Haematoxylin staining (blue).

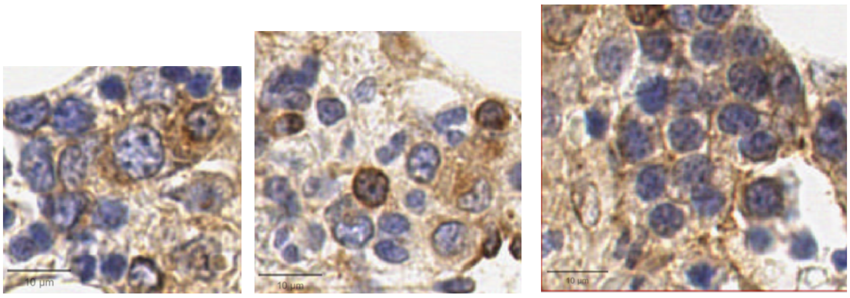

Basal NF-κB activation level definition

The definition of "basal levels" is intrinsic in the NF-κB system and has been tackled in one of our NF-κB first papers[3].

In unstimulated Mouse Embryo Fibroblasts in culture, the nucleus is almost devoid of NF-κB but the NCI values are never equal to zero and reveal a spontaneous oscillatory activity.

We believe that a sporadic minimal p65 activity might be required for the maintenance of the repressed state of the NF-κB signaling through basal resynthesis of IκB inhibitors, whose transcription is NF-κB dependent.

The threshold for activation is identified based on the results of basal NCI shown in Figure 1 for UT cells. In particular, most UT cells have a value below NCI=1.2, and for all the remaining situations/patients the value is higher. For this reason, we did not need a requisite number of samples to identify thresholds for basal levels in the MM cells, instead we analysed the distribution of p65 NCI in the UT population.

The concept of changes in the “basal levels” are now highlighted in Figure 3, where it is evident that the constant removal of secreted molecules leads to a homogeneous lower level of nuclear NF-κB in in both myeloma and stromal cells.

FIGURE 2

NF-κB dynamics in living MM cells are heterogeneous and active for many hours after stimulation

Deletions and duplications in genetic loci containing NF-kB signalling pathway genes have been reported, suggesting a selective advantage for PCs growth, although CNV tolerance has been hypothesized [4–6].
Before choosing a myeloma cell line for the in vivo analysis of NF-kB dynamics, we first asked whether the available MM lines contain the genetic heterogeneity documented by Lohr et al. in primary samples [6]. Indeed, a high variability in NF-kB regulatory genes dosage (Copy Number Variations, CNVs, Supplementary Figure 2a) was found in 24 out of 24 analysed cell lines.

Using an unsupervised hierarchical clustering, cell lines were grouped into three main groups with minimal, mild or extreme CNVs, green, blue, red boxes, respectively (Supplementary Figure 2a). As an example, MM1.S show only NFKBIA gene (IκBalpha repressor protein) haploinsufficiency.

We reasoned that such high CNVs in NF-κB regulatory genes could represent a Gain of Function (GoF) and positively or negatively affect NF-κB responses to the environment. Genes belonging to other signalling pathways, could impart additional deviations from physiologic responses.

Our mathematical model (Supplementary Figure 2b) in-silico explored NF-κB dynamics to inflammatory stimuli (e. g. TNF-α) in model cell lines bearing CNVs recapitulated by tuning the kinetic parameters affected by the mutations. The different responses are plotted as nuclear NF-κB fraction (y-axis) along time (x-axis, Supplementary Figure 2c, d). The physiological response predicted by the model in MEFs is reported as reference (cyan line and Ref [7,8]). In cells with NFKBIA haploinsufficiency, the model predicts a strong immediate peaked nuclear localization followed by a slow decrease in time without a complete clearing of the nucleus and therefore resolution of the response (MM.1S cells, red line) while normal cells would display oscillatory NF-kB dynamics (Blue line, Supplementary Figure 2c).

Our *in silico* analyses suggest that haploinsufficiency or hyperdiploidy in myeloma cell lines recapitulate end-point NF-κB deregulation as transcriptionally described in primary myeloma cells and lines[9] thus contributing to a hyper-responsiveness to inflammatory stimuli.

Why did we choose MM.1S to model MM?

We reasoned that a suitable cell model to provide a proof-of-concept for NF-κB dynamics alterations in living myeloma cells in the very early stage of the disease, would necessary bear minimal genetic alterations, both genome-wide and in the NF-κB regulatory pathway, as opposed to cells with skewed genotypes which might override NF-κB activity[10].

To validate model predictions, a panel of myeloma cell lines, including also normal EBV transformed B-cell lines, were screened for NF-κB nuclear translocation in response to inflammatory stimuli. Cells in culture were left either untreated or TNF-α stimulated for 30, 60, 90, 120 and 240 minutes, IF stained for p65 (Supplementary Figure 3 and 4) and imaged by confocal microscopy.
p65 translocation in JY and normal B cells was maximal in 30-60 min and decreased to basal level in 2-3 hrs (Supplementary Figure 3).

p65/NF-κB proteins in MM.1S cells are mainly, although not exclusively, located in the cytoplasm and relocalise to the nucleus upon a short TNF-α stimulation. However, the nuclear staining persists even after three hours stimulation, which is the physiological time by which p65 returns to a cytoplasmic location in most of the cell types analysed so far (Supplementary Figure 6).

U266 and OPM2 cells started to die soon after TNF-α stimulation, while RPMI8226 cells showed detectable p65 nuclear localization that was unaffected by TNF-α stimulation (Supplementary Figure 4).

From a genetic analysis (https://cancer.sanger.ac.uk/cell_lines and our analyses in Supplementary Figure 2a), the four cell lines (MM1.S < U266 < OPM2 < RPMI8226) contained progressively increasing numbers of mutations in NF-κB regulatory genes that might have been responsible for the complete deregulation. Our mathematical model predicts that gene mutations impact on the intensity of NF-κB activation and the extent of its nuclear persistence (Supplementary figure 2 and Supplementary Table 1).

Overall, we decided to use MM1.S cells because i) contain only one mutation in NF-κB regulatory genes, ii) 8 hr TNF-α stimulation does not affect their viability, iii) show a stronger response of NF-κB dynamics upon TNF-a, iv) in the mouse BM show low basal levels of activation, similar to the mild levels reported for patient samples.

FIGURE 4

A molecular crosstalk between MSCs and MM cells tunes NF-κB responses

To exclude the possible involvement of non-canonical NF-κB activities, which arise for stimulations of 16-24 hrs [11,12] , the inflammatory stimuli (TNF-α, IL-1β, IL-6, SDF-1, cocultures and conditioned media) have been applied for time-windows of 2-to-4 hours in the experiments from Figure 4 onwards.

FIGURE 5

Reciprocal myeloma-stroma crosstalk produces low-level NF-κB activation in a compact 3D ME

GSEA analyses highlighted the overall further activation of "TNF-α signalling via NF-κB" and "Interleukin signalling" in MM.1S cocultured with HS-5. The activation appears mild, although significant (FDR<0.02). The already high basal threshold of inflammatory gene transcripts in MM.1S cells might accounts for the small fold changes detected.

Indeed, the small Cluster 6 containing both MM.1S and HS-5 cells accounts for approximately 1% of the populations (lime green dots) and suggest that cocultures further activate the expression of high levels of NF-κB driven genes in minorities of highly responsive cells (Figure 4e, Supplementary Figure 11 h, k). In contrast, HS-5 cells upon MM.1S cocultures activate inflammatory transcriptional pathways with lower False Discovery Rates (< 0.0003).

FIGURE 6

IL-1β and paracrine signalling shape the myeloma BM ME and induce phenotypic changes

Maturation of MM cells

Immunoglobulin genes isotype switching and recombination are central events in plasmacell maturation [13] .However, maturation is progressive at population level and generates an heterogenous assortment of intermediate configurations. Therefore, from a transcriptional standpoint it is possible to capture cells with an intermediate phenotype by scRNAseq upon specific stimuli (e.g. coculture with stromal cells). Indeed, MM1.S cells are stabilised from an MM patient, therefore, we expect to find all the possible maturation levels compatible with in vitro cell culture.

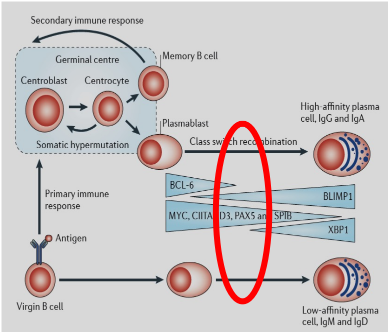

Cell crowding in the BM facilitates MM-MSCs crosstalk.

The image below contains an example of a possible crosstalk between two MSCs and a MM cell in the BM of a myeloma patient.
The immunofluorescence staining of a BM section from Pt.1 shows p65 (grey), CD44 (MSC marker, green), CXCL12 (one of the CKs mainly secreted by MSCs (red) and nuclei in blue as indicated. Scale bar: 50 µm.
The two small images below are enlargements of the squared areas in the large pictures above. Scale bar: 10 µm. Grey filled arrow points to a MM cell showing some p65 in the nucleus and very closed to two MSCs (green + red= yellow, and yellow arrow).
The grey empty arrow points to a MM cell with a cytoplasmic localization of p65.

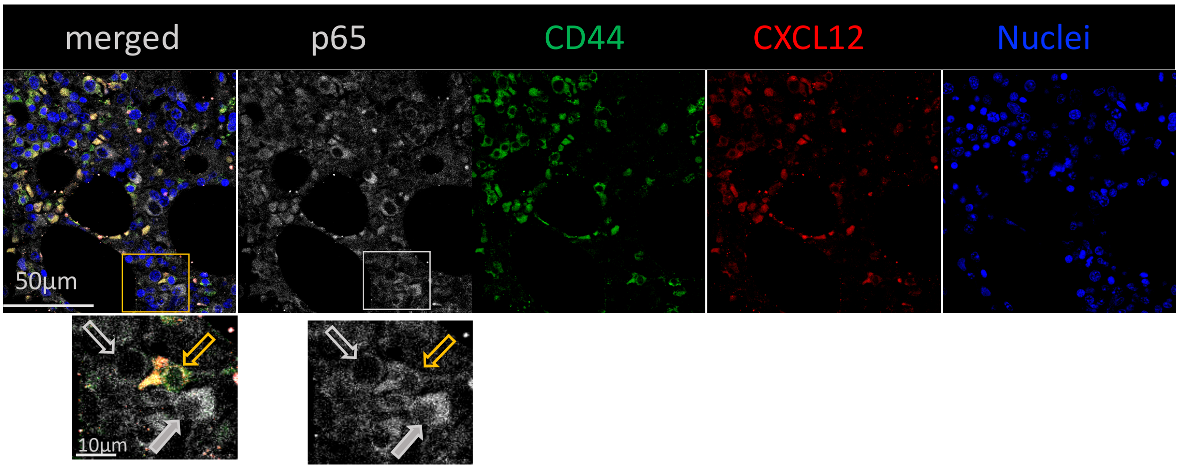

BM regions enriched in IL-1β secreting cells show nuclear NF-κB in myeloma cells.
Three representative images from BM samples from 3 patients out of nine analysed, stained with anti p65/NF-κB (green) and anti IL-1β (red), and nuclei are counterstained with Hoechst33342. Scale bar 10µm. Each field contains IL-1β positive cells and some myeloma cells with p65 nuclear localization (red and green arrows, respectively).

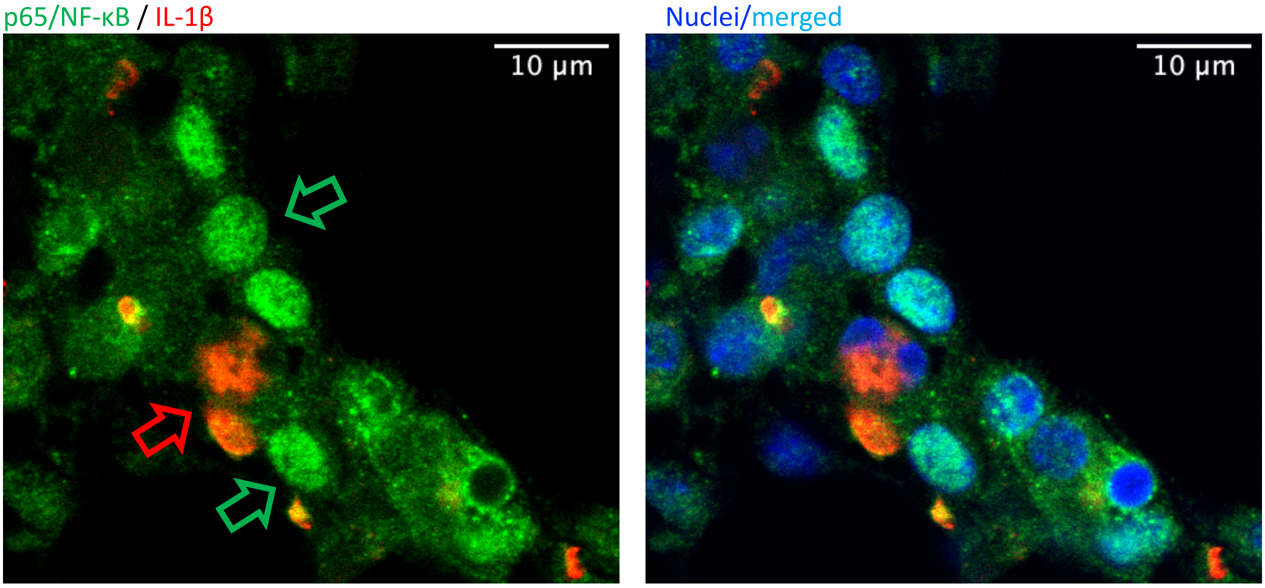

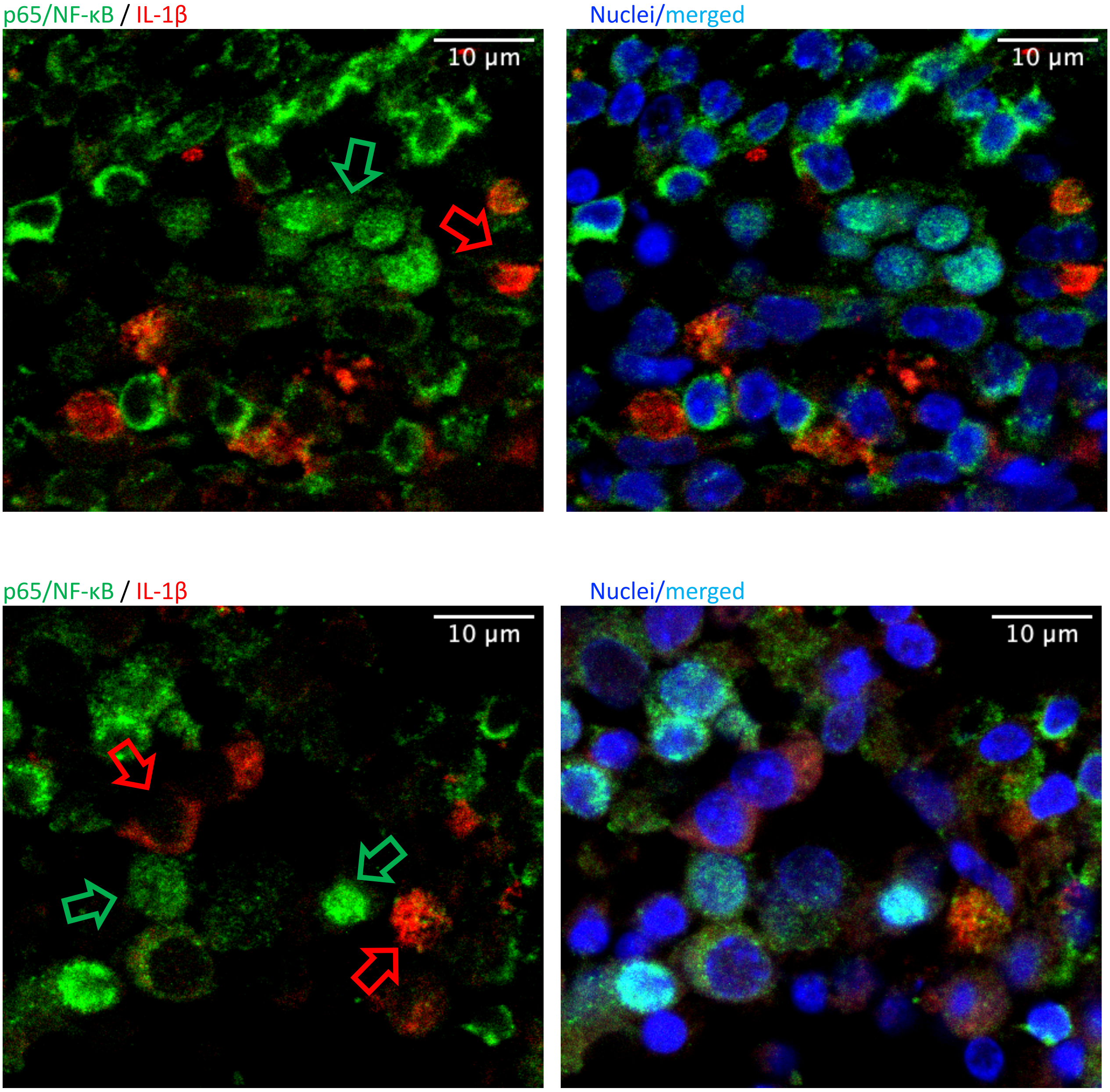

REFERENCES

1 Greenstein S, Krett NL, Kurosawa Y, Ma C, Chauhan D, Hideshima T *et al.* Characterization of the MM.1 human multiple myeloma (MM) cell lines: A model system to elucidate the characteristics, behavior, and signaling of steroid-sensitive and -resistant MM cells. Exp Hematol. 2003; **31**: 271–282.

2 Kalderon AE, Bogaars HA, Diamond I, Cummings FJ, Kaplan SR, Calabresi P. Ultrastructure of myeloma cells in a case with crystalcryoglobulinemia. *Cancer* 1977; **39**: 1475–81.

3 Zambrano S, Bianchi ME, Agresti A. High-Throughput Analysis of NF-kappaB Dynamics in Single Cells Reveals Basal Nuclear Localization of NF-kappaB and Spontaneous Activation of Oscillations. *PLoS One* 2014; **9**: e90104.

4 Chapman MA, Lawrence MS, Keats JJ, Cibulskis K, Sougnez C, Schinzel AC *et al.* Initial genome sequencing and analysis of multiple myeloma. *Nature* 2011; **471**: 467–472.

5 Lohr JG, Stojanov P, Carter SL, Cruz-Gordillo P, Lawrence MS, Auclair D *et al.* Widespread genetic heterogeneity in multiple myeloma: implications for targeted therapy. *Cancer Cell* 2014; **25**: 91–101.
6 Lohr JG, Kim S, Gould J, Knoechel B, Drier Y, Cotton MJ *et al.* Genetic interrogation of circulating multiple myeloma cells at single-cell resolution. *Sci Transl Med* 2016; **8**: 363ra147-363ra147.
7 Zambrano S, Bianchi ME, Agresti A. A simple model of NF-kappaB dynamics reproduces experimental observations. *J Theor Biol* 2014; **347**: 44–53.
8 Zambrano S, Bianchi ME, Agresti A. High-throughput analysis of NF-kB dynamics in single cells reveals basal nuclear localization of NF-kB and spontaneous activation of oscillations. *PLoS One* 2014; **9**. doi:10.1371/journal.pone.0090104.
9 Annunziata CM, Davis RE, Demchenko Y, Bellamy W, Gabrea A, Zhan F *et al.* Frequent Engagement of the Classical and Alternative NF-κB Pathways by Diverse Genetic Abnormalities in Multiple Myeloma. *Cancer Cell* 2007; **12**: 115–130.
10 Birchler JA, Veitia RA. Gene balance hypothesis: Connecting issues of dosage sensitivity across biological disciplines. *Proceedings of the National Academy of Sciences* 2012; **109**: 14746–14753.
11 Yılmaz ZB, Kofahl B, Beaudette P, Baum K, Ipenberg I, Weih F *et al.* Quantitative Dissection and Modeling of the NF-κB p100-p105 Module Reveals Interdependent Precursor Proteolysis. *Cell Rep* 2014; **9**: 1756–1769.
12 Meier-Soelch J, Mayr-Buro C, Juli J, Leib L, Linne U, Dreute J *et al.* Monitoring the Levels of Cellular NF-κB Activation States. *Cancers (Basel)* 2021; **13**: 5351.
13 Morgan GJ, Walker BA, Davies FE. The genetic architecture of multiple myeloma. *Nat Rev Cancer* 2012; **12**: 335–348.

Supplementary Table 1: MM1.S mutations

Supplementary Table 2 (Cluster_markers): list of genes characterizing each cluster in Figure 4e.

Supplementary Table 3: *Summary of cell number, median values in each experimental plot throughout the paper.*
